# Supplementary figures and images for: An Sfi1-like centrin-interacting centriolar plaque protein affects nuclear microtubule homeostasis
Source: PLoS Pathog. 2023 May 2;19(5):e1011325. doi: 10.1371/journal.ppat.1011325 (PMC10180636; doi:10.1371/journal.ppat.1011325)

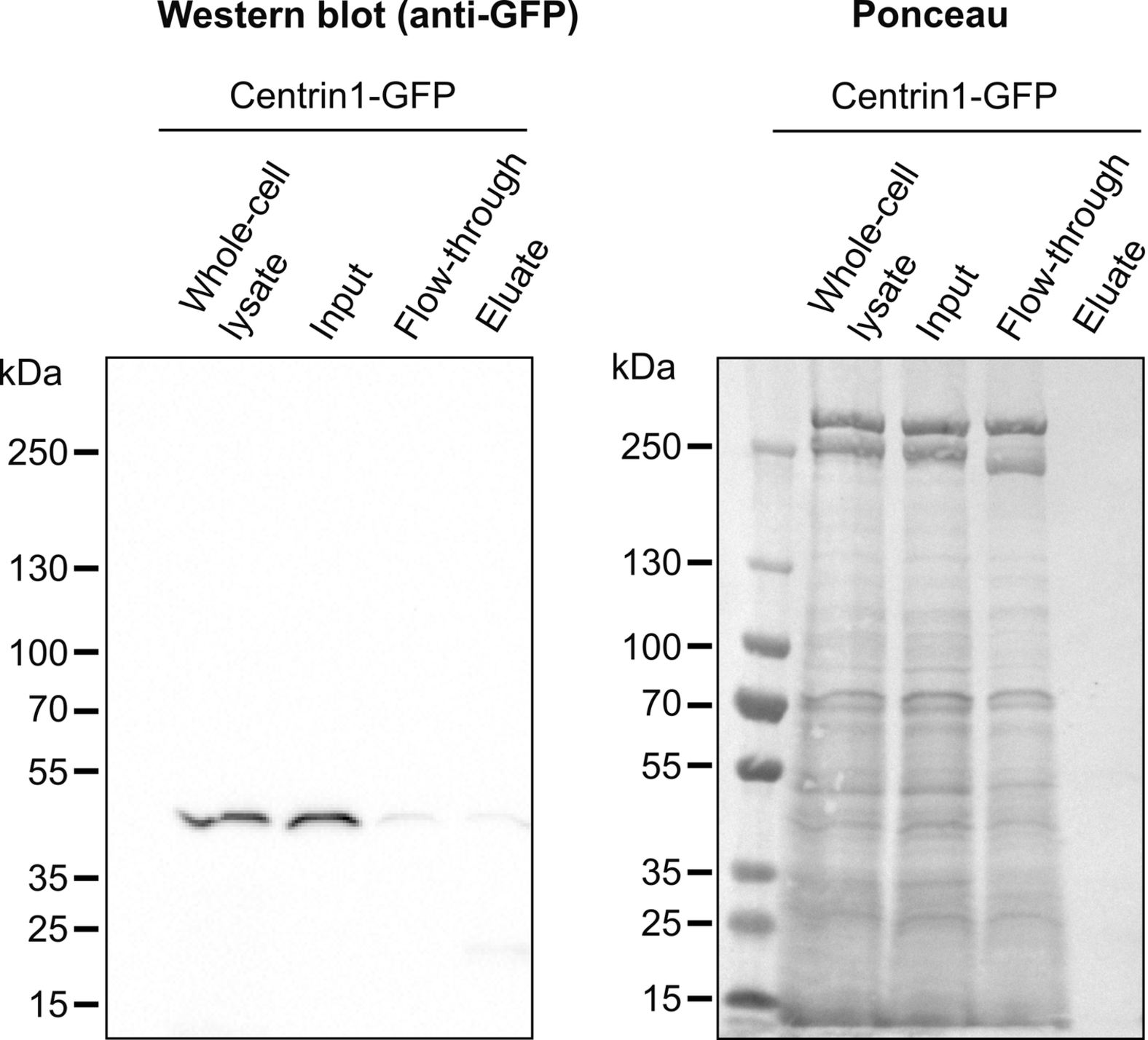

Supplement: S1 Fig — “Whole-cell lysate”, “input”, “flow-through” and “eluate” sample fractions were taken at different steps of PfCentrin1-GFP co-immunoprecipitation as described in the methods section. Per lane, protein lysate of 1 x 10^7^ parasite cells was loaded (number of infected red blood cells per ml determined before Saponin-lysis during cell harvesting). Size of the bands (circa 47 kDa) detected by the anti-GFP antibody in all lanes of the western blot corresponds to the molecular weight of PfCentrin1 (19.6 kDa) tagged with GFP (26.9 kDa). In the eluate fraction some degradation of PfCen1-GFP could be observed. Please note that the weak Centrin1-GFP band in the eluate might be due to loss of protein lysate during the individual (washing) steps of the IP. This is consistent with absence of protein signal (neither for Centrin1-GFP nor for unspecific proteins) in the eluate lane by Ponceau staining. (TIF) [file ppat.1011325.s001.tif]

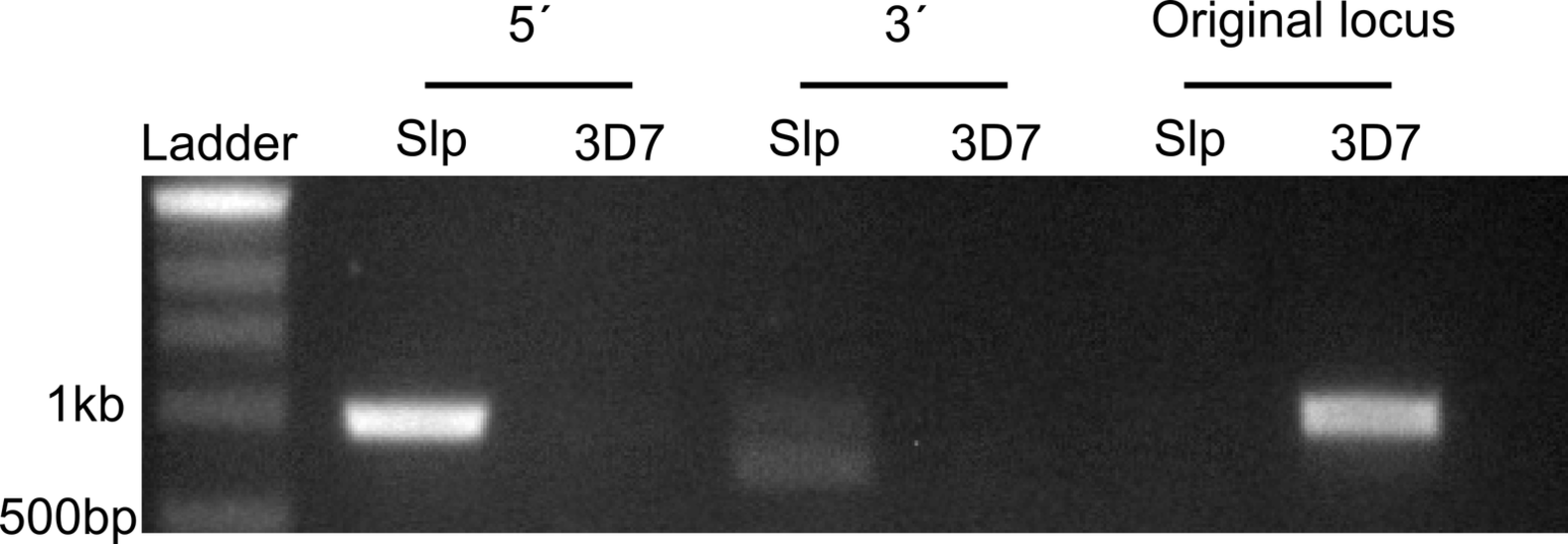

Supplement: S2 Fig — PCR of PfSlp-GFP transfected cells and 3D7 wildtype cells targeting 5´ and 3´ integrations to validate complete integration of the GFP-glmS tag to endogenous PfSlp. Control PCR targeting the unaltered locus of PfSlp in the 3D7 wild type strain confirms complete integration and modification of the endogenous locus. (TIF) [file ppat.1011325.s002.tif]

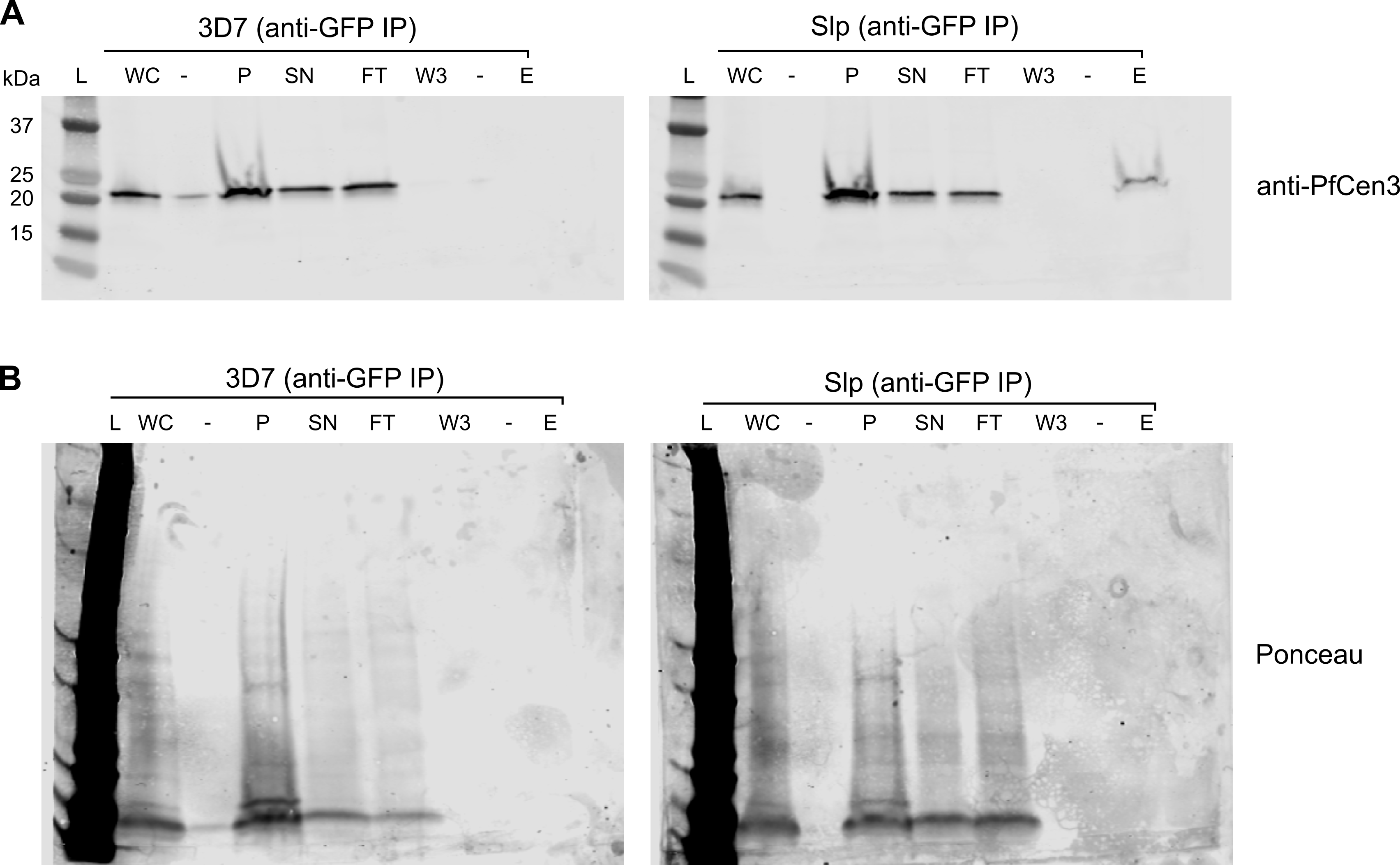

Supplement: S3 Fig — A) Western blot analysis of co-immunoprecipitation using anti-GFP beads on 3D7 wild type whole cell lysate (‘WC’) on the left and Slp ‘WC’ on the right. Centrin was detected by anti-PfCen3 antibody. Per lane, protein lysate of 4 x 10^7^ late stage parasite cells were loaded (number of infected red blood cells per ml determined before Saponin-lysis during cell harvesting). Other lanes contain pellet (P), supernatant (SN), flow-through (FT), third wash (W3), and eluate (E) sample fractions, which were taken at different steps of co-immunoprecipitation as described in the methods section. ‘-‘ designates empty lanes. Size of the bands in all lanes correspond to the molecular weight of centrins (~20 kDa). B) Ponceau staining shows absence of protein signal in the eluate lane. (TIF) [file ppat.1011325.s003.tif]

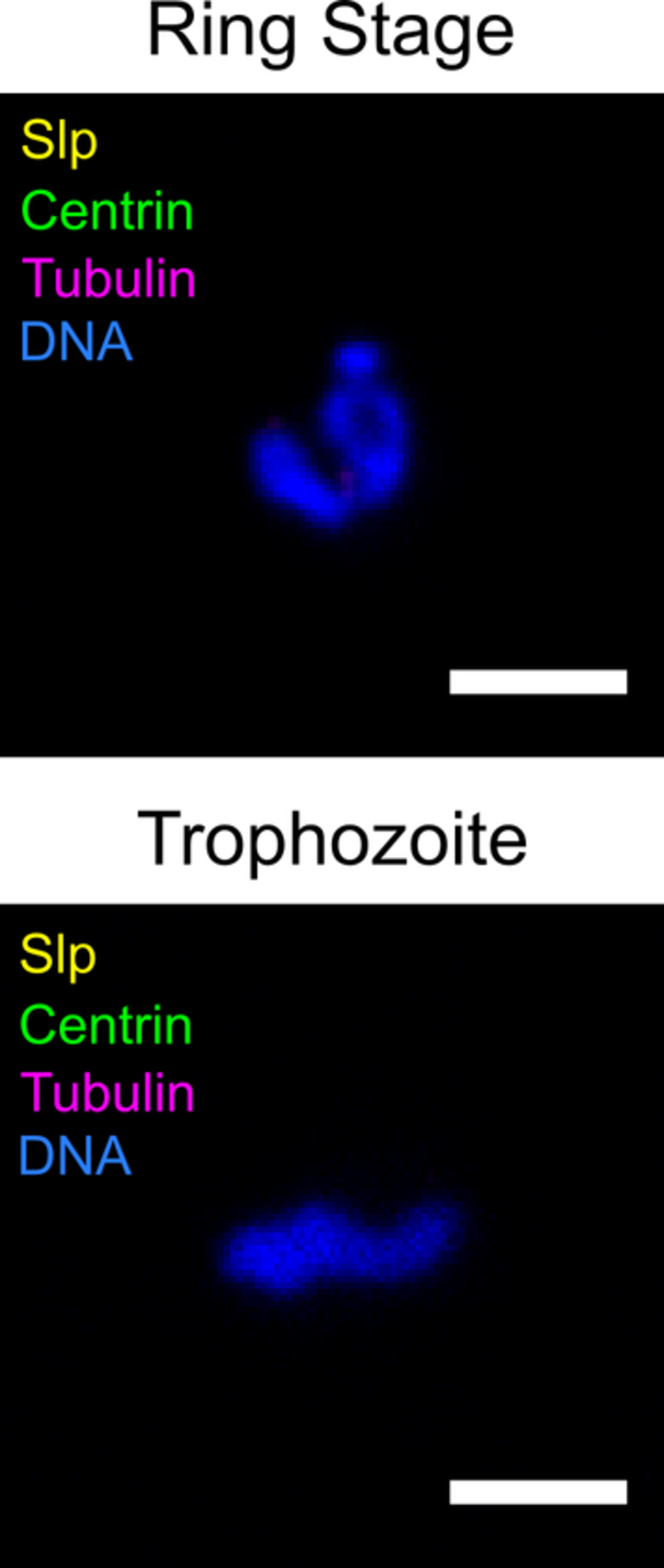

Supplement: S4 Fig — Confocal microscopy images of immunofluorescence staining of ring stage parasites and trophozoites expressing endogenously tagged PfSlp-GFP using anti-centrin, anti-tubulin and anti-GFP antibodies. DNA stained with Hoechst. Maximum intensity projections are shown. Scale bars, 1.5 μm. (TIF) [file ppat.1011325.s004.tif]

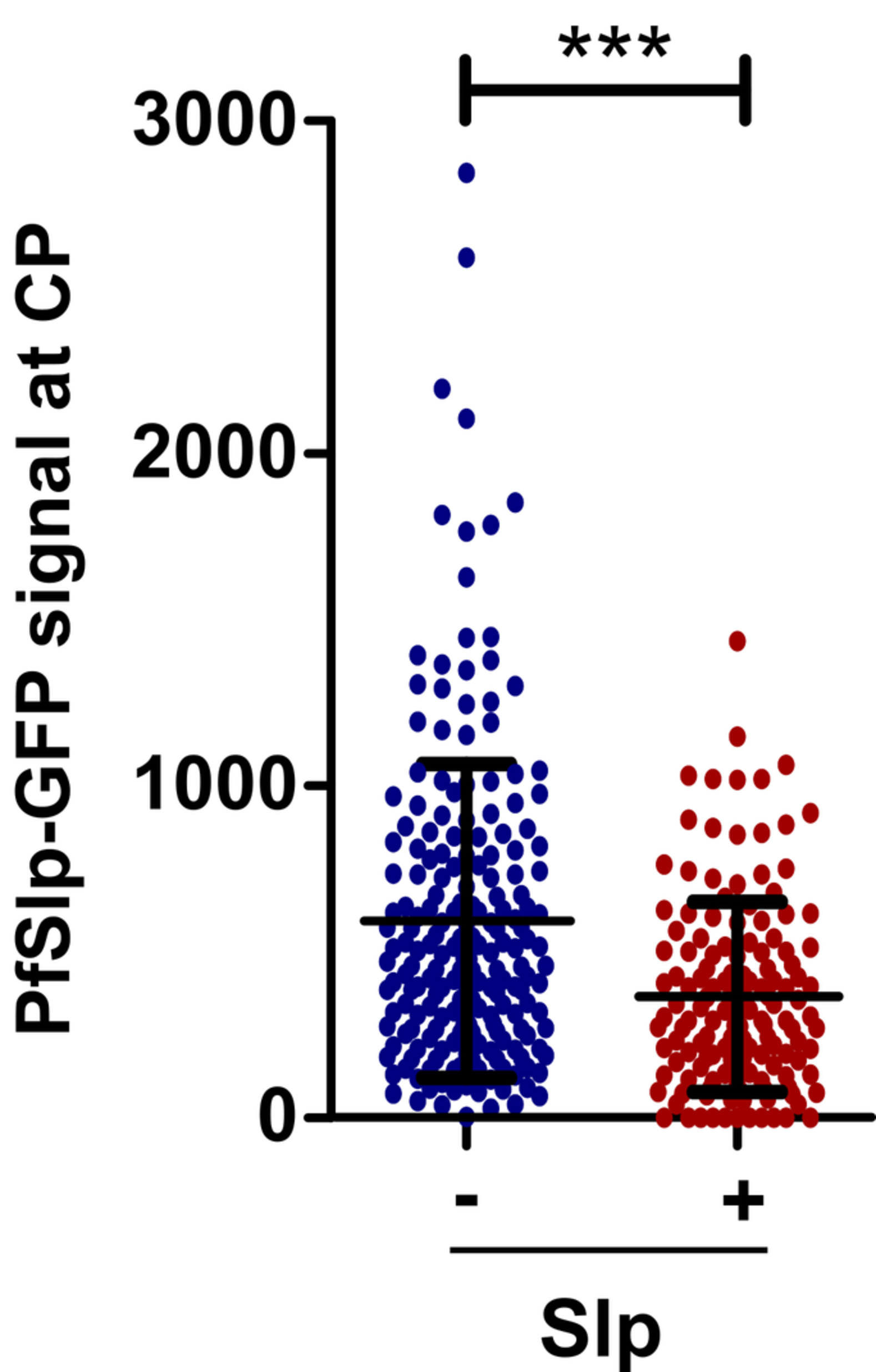

Supplement: S5 Fig — Relative fluorescence signal intensity of PfSlp-GFP signal at the centriolar plaque was measured in +/-GlcN Slp schizont parasites with up to 10 nuclei, immunostained as in Fig 1D. Means generated from three biological replicates (N = 3). SEM is shown. Statistical analysis by t-test with Welch´s correction. ***: p<0.0001. (TIF) [file ppat.1011325.s005.tif]

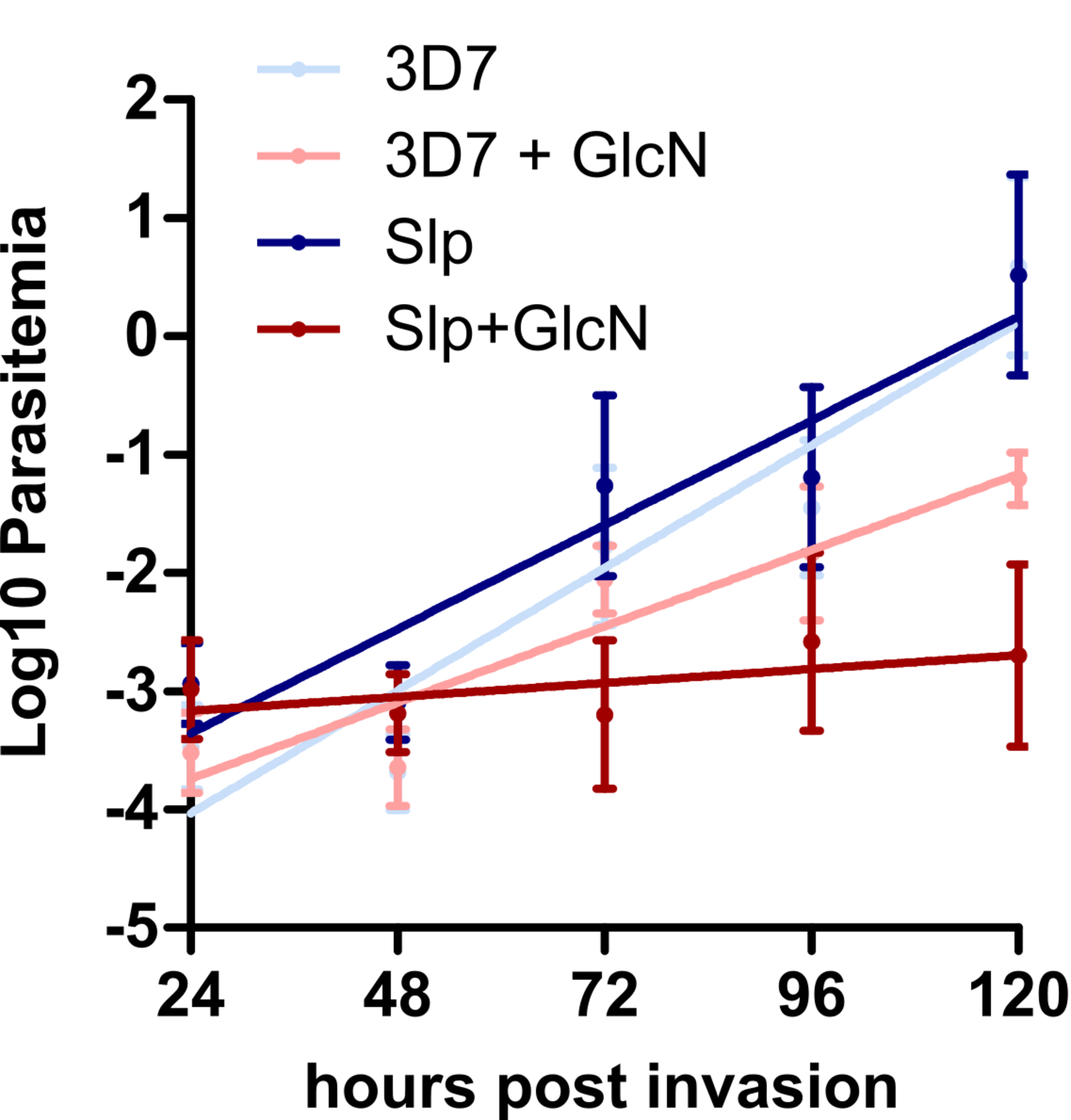

Supplement: S6 Fig — Growth curves of 3D7 wild type and Slp strain +/- GlcN. Parasitemia was measured via flow cytometry after SYBR-Green staining and fixation for 5 days after treatment. Log10 of parasitemia is plotted to highlight differences in slope. All means generated from three biological replicates (N = 3). SEM is shown. (TIF) [file ppat.1011325.s006.tif]

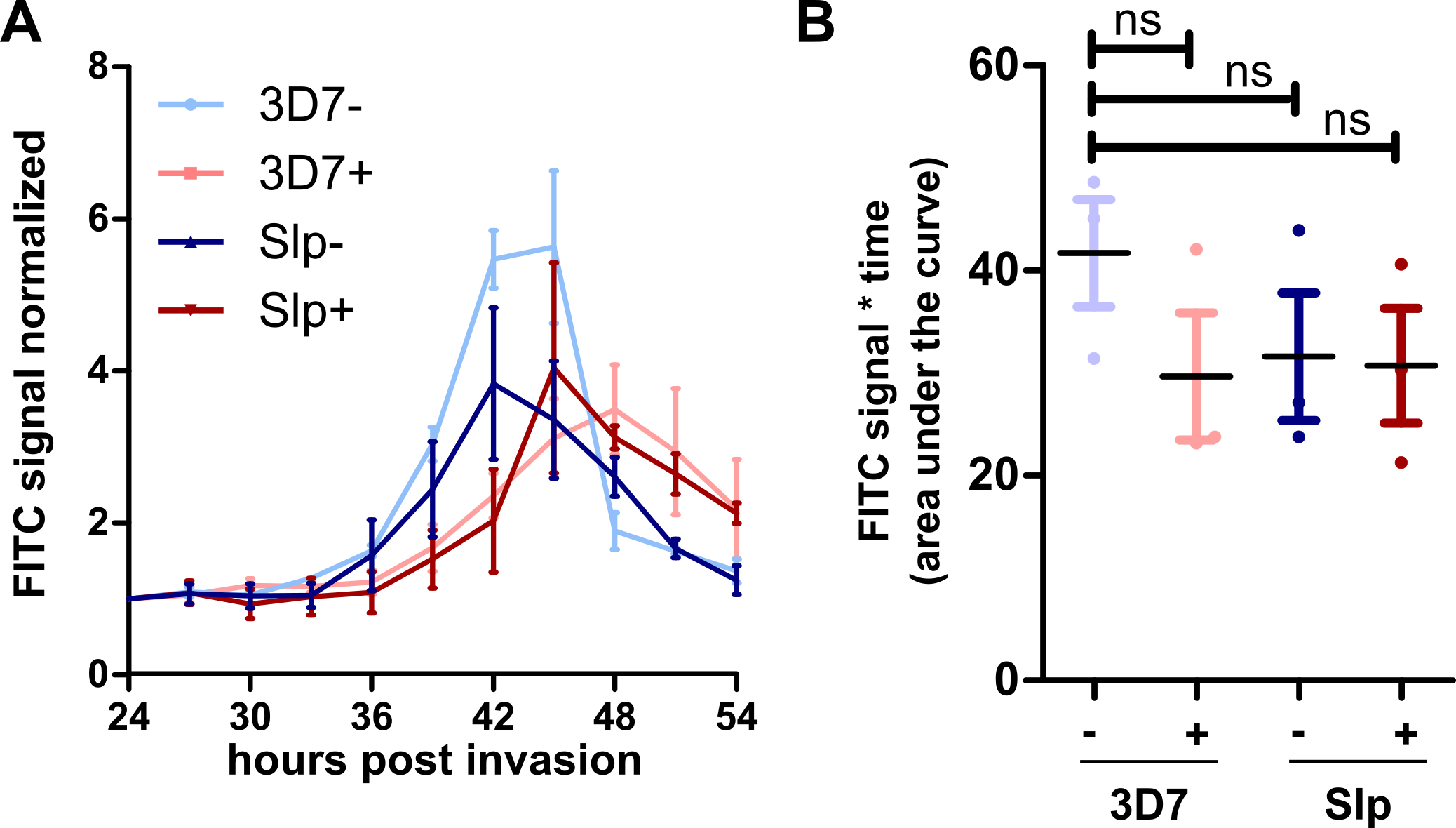

Supplement: S7 Fig — A) Flow cytometry analysis of SYBR-Green stained and RNase treated 3D7 and Slp parasites +/-GlcN indicating changes in DNA replication. Mean intensity normalized to 1 at 24 hpi. B) Area under the curve for three replicas of each condition shown in A) calculated in Prism. Statistical analysis: t-test with Welch´s correction. (TIF) [file ppat.1011325.s007.tif]

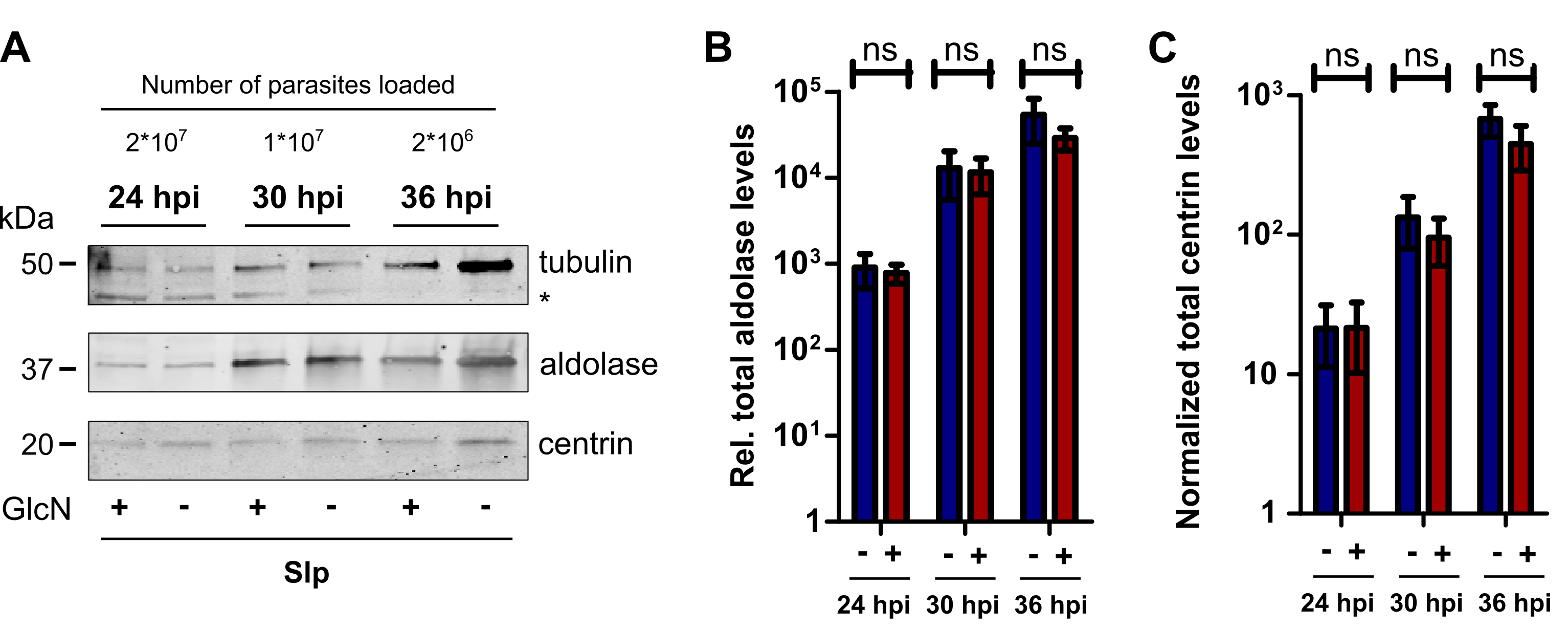

Supplement: S8 Fig — A) Synchronized Slp parasites +/- GlcN were harvested at 24, 30 or 36hpi, and SDS-PAGE of 2*107, 1*107 or 2*106 parasites per lane respectively was performed. After blotting, the blot was cut at 25 kDa. The upper part was incubated with rabbit-anti-aldolase and mouse anti-tubulin primary antibodies, and anti-rabbit 680RD and anti-mouse 800CW secondaries, while the lower part was incubated with rabbit anti-Centrin3 primary and anti-rabbit 800CW secondary antibodies. Band at 50 kDa corresponds to the molecular weight of tubulin, a lower unspecific band is marked as (*). Band at 37 kDa and 20 kDa correspond to aldolase and centrin size respectively. Unspecific band was not included in measurement B) Quantification of aldolase protein signal after correcting for equal parasite number. C) Quantification of centrin protein level after correcting to equal parasite number, normalized to aldolase signal. Slp -GlcN (blue). Slp +GlcN (red). All means generated from three biological replicates. Statistical analyses: t-test with Welch´s correction. ns: p>0.05. (TIF) [file ppat.1011325.s008.tif]

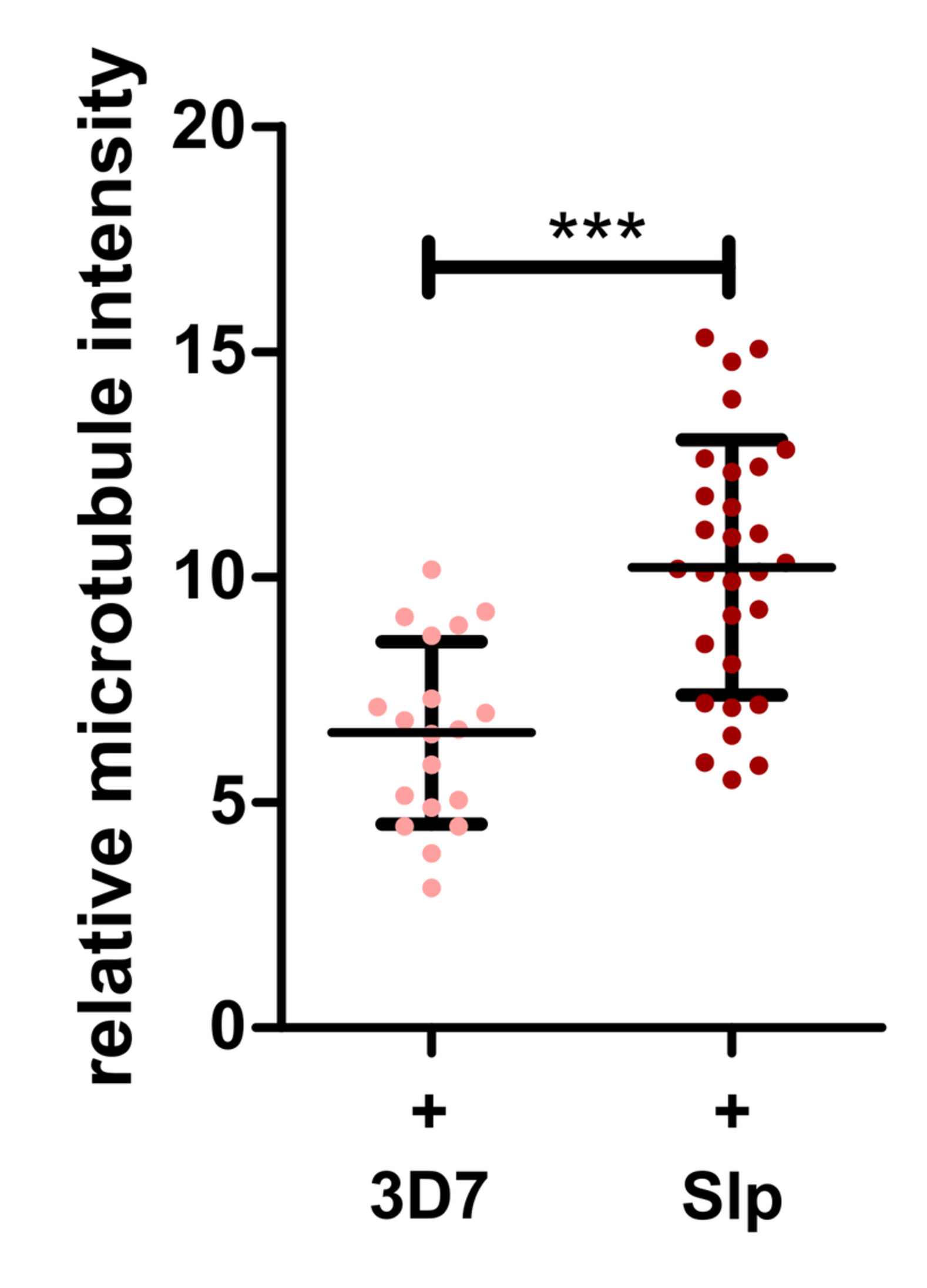

Supplement: S9 Fig — Movies of SPY555-Tubulin labelled 3D7 +GlcN and Slp +GlcN treated cells acquired in the same imaging session using identic settings. For quantification the first timeframe showing a defined mitotic spindle was selected and the relative microtubule signal intensity was quantified using average intensity projections of image slices containing the spindle microtubule signal. Means generated from three independent imaging sessions (N = 3). SD is shown. Statistical analysis by t-test with Welch´s correction. ***: p<0.0001. (TIF) [file ppat.1011325.s009.tif]
