# Supplementary material for: An Sfi1-like centrin-interacting centriolar plaque protein affects nuclear microtubule homeostasis
Source: PLoS Pathog. 2023 May 2;19(5):e1011325. doi: 10.1371/journal.ppat.1011325 (PMC10180636; doi:10.1371/journal.ppat.1011325)
Supplement: S1 Table — All proteins identified from the non-crosslinked PfCentrin1-GFP co-immunoprecipitation samples are listed with peptide counts. Only Centrin-1, Centrin-3, and PfSlp (highlighted in green) were not detectable in a GFP only control pull down done with the same protocol used in a previous study by Balestra et al. 2021 [50]. (PDF) [file ppat.1011325.s010.pdf]

Gene ID	Identified Proteins	M.W.	Peptide count	GFP control pull down (Balestra et al. 2021) [1]
**PF3D7_0107000**	**Centrin-1**	**20 kDa**	**24**	**not identified in GFP control**
PF3D7_1357000	Elongation factor 1-alpha	49 kDa	5	identified in GFP control
PF3D7_1462800	Glyceraldehyde-3-phosphate dehydrogenase	37 kDa	5	identified in GFP control
PF3D7_0818900	Cluster of Heat shock protein 70	74 kDa	5	identified in GFP control
**PF3D7_1027700**	**Centrin-3**	**21 kDa**	**4**	**not identified in GFP control**
PF3D7_1117700	GTP-binding nuclear protein	25 kDa	3	identified in GFP control
**PF3D7_0710000**	**Conserved protein, unknown function**	**407 kDa**	**3**	**not identified in GFP control**
PF3D7_0610400	Histone H3	15 kDa	2	identified in GFP control
PF3D7_0719600	60S ribosomal protein L11a, putative	20 kDa	2	identified in GFP control
PF3D7_0708400	Heat shock protein 90	86 kDa	2	identified in GFP control
PF3D7_1116800	Heat shock protein 101	103 kDa	2	identified in GFP control

S1 Table. Mass spectrometry analysis of Centrin Co-IP reveals two specific interaction partners. All proteins identified from the non-crosslinked PfCentrin1-GFP co-immunoprecipitation samples are listed with peptide counts. Only Centrin-1, Centrin-3, and PfSlp (highlighted in green) were not detectable in a GFP only control pull down done with the same protocol used in a previous study by Balestra et al. 2021 [1].

Reference

1. Balestra AC, Koussis K, Klages N, Howell SA, Flynn HR, Bantscheff M, et al. Ca 2+ signals critical for egress and gametogenesis in malaria parasites depend on a multipass membrane protein that interacts with PKG. Sci Adv. 2021;7: eabe5396. doi:10.1126/sciadv.abe5396.
