# Supplementary material for: An Sfi1-like centrin-interacting centriolar plaque protein affects nuclear microtubule homeostasis
Source: PLoS Pathog. 2023 May 2;19(5):e1011325. doi: 10.1371/journal.ppat.1011325 (PMC10180636; doi:10.1371/journal.ppat.1011325)
Supplement: S2 Table — (PDF) [file ppat.1011325.s011.pdf]

Primer designation	Sequence
1: Slp cDNA fw 1	GAAGACGATGTTGAGGAGGGG
2: Slp cDNA rev 1	TCGTCGTTATGGACATCCTCT
3:Slp cDNA fw 2	AGGTGAAAGTATAAGCGGTCAGG
4: Slp cDNA rev 2	CCCCTCCTCAACATCGTCTT
5: Serine tRNA ligase fw	AAGTAGCAGGTCATCGTGGTT
6: Serine tRNA ligase rev	TTCGGCACATTCTTCCATAA
7: gDNA 5´ integration fw	CACCACATCTTCATAACTCTTCAGG
8: gDNA 5´ integration rev	GCATCACCTTCACCCTCTCC
9: gDNA 3´ int fw	GAGCGGATAACAATTTCAC
10: gDNA 3´ int rev	CAAAACATGTTTACATTATTGACAAGG
7: gDNA original locus fw	CACCACATCTTCATAACTCTTCAGG
10: gDNA original locus rev	CAAAACATGTTTACATTATTGACAAGG

S2 Table. List of primers used in this study.
