# Supplementary material for: An Sfi1-like centrin-interacting centriolar plaque protein affects nuclear microtubule homeostasis
Source: PLoS Pathog. 2023 May 2;19(5):e1011325. doi: 10.1371/journal.ppat.1011325 (PMC10180636; doi:10.1371/journal.ppat.1011325)
Supplement: S3 Table — (PDF) [file ppat.1011325.s012.pdf]

Antibody	Species	Dilution	Source
anti-alpha-tubulin B-5-1-2, monoclonal	mouse	1:500	Sigma
Anti-tubulin [YOL1/34]	rat	1:200	Abcam
anti-PfCentrin3, polyclonal	rabbit	1:500 (1:1000 for WB)	Simon et al., 2021
Anti-GFP 3E6, monoclonal	mouse	1:200	Thermo
Anti-EGFP [F56-6A1.2.3]	mouse	1:50	Abcam
Anti GFP (11814460001, Western blot)	mouse	1:2000	Roche
HRP Anti- PfAldolase antibody (ab38905)	rabbit	1:1000	Abcam
anti-rat-Alexa 488	Goat	1:1000*	Sigma
anti-mouse-Alexa 568	Goat	1:1000	Sigma
anti-mouse-Atto647	Goat	1:1000*	Sigma
anti-rabbit-Atto594	Goat	1:1000*	Sigma
anti-rabbit-Atto647	Goat	1:1000	Sigma
Anti-mouse IgGHRP (A5278)	Goat	1:3000	Sigma
IRDye 800CW anti-Mouse IgG (H + L)	Goat	1:10.000	LI-COR
IRDye 680RD anti-Rabbit IgG (H + L)	Goat	1:10.000	LI-COR
IRDye 800CW anti-Rabbit IgG (H + L)	Goat	1:10.000	LI-COR
**Dye**		**Dilution**	**Source**
SPY555-Tubulin (SC203)	-	1:2000	Spirochrome
5-SiR-Hoechst	-	20 nM	G. Lukinavicius
Hoechst33342	-	1:1000	Thermo

S3 Table. Antibodies and dyes used in this study. Starred (*) indicates dilutions for IFAs imaged by confocal microscopy; for STED, those antibodies were used at 1:200.

Reference

1. Simon CS, Funaya C, Bauer J, Voβ Y, Machado M, Penning A, et al. An extended DNA-free intranuclear compartment organizes centrosome microtubules in malaria parasites. Life Sci Alliance. 2021;4: e202101199. doi:10.26508/lsa.202101199
