# Supplementary material for: An Sfi1-like centrin-interacting centriolar plaque protein affects nuclear microtubule homeostasis
Source: PLoS Pathog. 2023 May 2;19(5):e1011325. doi: 10.1371/journal.ppat.1011325 (PMC10180636; doi:10.1371/journal.ppat.1011325)
Supplement: Attachment — Submitted filename: 129260_1_rebuttal_2551909_rnk1rs.pdf [file ppat.1011325.s019.pdf]

Full Revision

Review COMMONS

Manuscript number: RC-2022-01614

Corresponding author(s): Julien, Guizetti

1. General Statements [optional]

We want to thank the editors and reviewers for taking the time to evaluate our manuscript and herewith present a full revision. Please find details concerning the goal of the study in the cover letter.

2. Point-by-point description of the revisions

Reviewer #1 (Evidence, reproducibility and clarity (Required)):

The manuscript "An Sfi1-like centrin interacting centriolar plaque protein affects nuclear microtubule homeostasis" by Wenz and co-authors describes the detection and analysis of the Sfi1-like protein in apicomplexan parasite Plasmodium falciparum. The authors examined the protein localization and function in asexual stages during parasite replication in the red blood cells. The authors detected PfSlp in the PfCentrin1 pulldown, created PfSlp conditional knockdown strain, and evaluated growth and morphological deficiencies associated with the PfSlp deficiency. The study's primary finding is that PfSlp inhibits the extension of nuclear MTs.

Major comments

The key conclusion is appropriate but is poorly supported by experimental evidence. The transitional, experiment-to-experiment conclusions are preliminary and may require additional experiments. The authors did not present a convincing model of the PfSlp1 function in mitosis.

We appreciate the reviewer's evaluation that our key conclusions are appropriate, but also have taken some of the valid comments below into account and added some conclusive experimental data and partly modified the choice of words when interpreting the data. We are now fully convinced that our conclusions are appropriate and supported by experimental evidence. To understand the function of PfSlp, which was described for the first time in this study, precisely will require a more detailed model of the still very much understudied malaria parasite centrosome and will be the subject of future inquiries.

If PfSlp inhibits the MT polymerization, then the PfSlp reduction should lead to an extension of the bipolar spindle, which is partly supported by longer MTs in the hemispindles. How is the excess of the nuclear MTs prevent the spindle resolution in anaphase?

Intranuclear hemispindle microtubules are indeed elongated. Increased microtubule polymerization does not necessary lead to an increased spindle length but could just as well promote the nucleation of multiple short microtubules or increase overlap between antiparallel

microtubules. We, however, want to emphasize that our key conclusion is that PfSlp is implicated in the regulation of nuclear tubulin levels, rather than “inhibits extension of nuclear MT”. In our view this is an important distinction since microtubule misorganization is merely a consequence of changing nuclear tubulin levels. At no point we want to suggest that PfSlp somehow directly inhibits polymerization of microtubules and therefore did not provide any specific evidence. The fact that PfSlp and microtubules are in different compartments underlines this. Yet, we have noted that our abstract uses the word polymerization. Although we mention that it occurs as a consequence of increased tubulin concentration, which thermodynamically favors microtubule polymerization, we acknowledge that this could be misleading and removed this term (line 30). Concerning how the excess nuclear MTs prevent anaphase spindle resolution we propose several explanations in the discussion (lines 381ff). **All line numbers refer to document with “tracked changes”.**

Fig 4C misrepresents mitotic phases: bipolar spindle should be broken into two in anaphase, while the drawing shows one elongated spindle connecting two poles.

Indeed, we frequently observed, anaphase spindles being “split” ourselves (Simon et al. LSA, 2021, Fig. 2A). Although sometimes we would see one elongated spindle and sometimes more than two as in Liffner et al. 2021 Fig. 3A. For simplicity we only drew one elongated interpolar microtubule bundle but have now corrected this for more accurate representation.

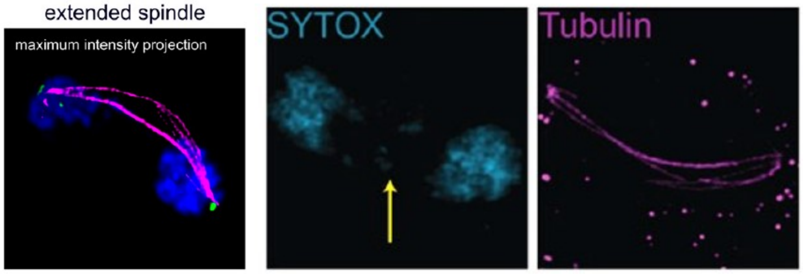

The authors should correct the use of terminology. Throughout the manuscripts, the parasite division stages are called life stages. Life stages are merozoites, gametocytes, ookinetes, sporozoites, etc. The division stages apply to a single life stage and, in the case of schizogony, are rings, trophozoites, and schizonts.

We once falsely referred to life cycle in line 182 when we should have referred to the intraerythrocytic development cycle. The paragraph using the incorrect wording was removed in the revision.

Please, note that schizogony does not follow the ring and trophozoite stages (line 119); it includes them as the distinctive morphological stages of one round of schizogony. The cell cycle terminology is incorrectly applied.

We have the impression that the usage of the term schizogony is rather “fluid” in that it is occasionally also employed to just the describe the phase where DNA replication, nuclear division, and cytokinesis occur (hence schizont stage), but we clearly note the more canonical

use as equivalent of the asexual intraerythrocytic development cycle as whole. We modified the terminology accordingly (e.g. by employing “schizont stage”) lines 43, 142, 184, 238, 265.

What is the "mitotic spindle stage," "mitotic spindle nuclei, "or "mitotic spindle duration" (Fig. 4B)?

It has now been conclusively demonstrated that nuclei go through independent nuclear cycles with different morphological stages (Simon et al. 2021 LSA, Klaus et al. 2022 Sci Advances). Hence, we use the term “mitotic spindle stage” to contrast it with the “hemispindle stage”, which can be morphologically distinguished using microtubules as a marker and occurs just prior to S-Phase. Consequently, “mitotic spindle nuclei” are nuclei in the “mitotic spindle stage”. “mitotic spindle duration” designates the time nuclei spend in that stage i.e. from hemispindle collapse until anaphase spindle elongation. We have adjusted and more accurately defined the terminology throughout the text and complemented Fig. 1A for clarity.

Minor comments

The PfSlp knockdown is inefficient: the 55% reduction at the RNA level translates into a minor change at the protein level (Fig.2 and S4). The evaluation of the protein changes should be done by western blot analysis with appropriate controls. The intensity of the IFA signal (used in the study) changes depending on the focal plane, as seen in Fig 1D.

Due to the exceptionally big size of PfSlp of around 407 kDa and the low expression levels western blot analysis was not feasible in our hands. For quantification of the IFA signal we used image projections and background subtraction to integrate the signal of the full z-stack containing the entire cell and our measurement was therefore independent of the focal plane. We have now described this a bit more thoroughly in the methods section (lines 620ff). The change in signal as measured by IFA is still clearly significant and shows a reduction of about 45%, which is coherent with the reduction of 55% found by RNA analysis and ultimately results in a specific phenotype.

Growth defects of the PfSlp KD: It is unclear what causes the reduced parasitemia of the GlcN untreated Slp parasites (Fig. 2C and D).

A likely explanation is that the C-terminal tagging of PfSlp already slightly impairs the function of the protein causing a mild growth phenotype that is not observed in wild type although it is not statistically significant (Fig. 2C). Importantly, the reproduced analysis of parasite growth, shown as multiplication rate in Fig. 2C (and growth curve in Fig. S6) now more clearly demonstrates that when normalizing for GlcN treatment and GFP-glms tagging (“3D7 corr.”) the growth defect is still significant and can therefore be attributed to Slp KD and not to tagging or GlcN treatment addition, which on their own do not cause a significant phenotype.

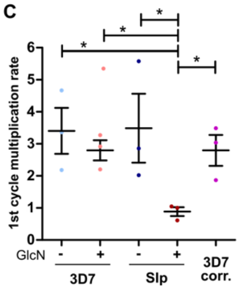

To conclude that the kinetics of DNA replication is affected, the authors will need to perform the real-time measurements of DNA replication forks.

We thank the reviewer for pointing this out and removed the term “kinetics” (line 182, 269).

The presented data supports that fewer S/M rounds were performed by PfSlp lacking parasites but gives no way to determine whether the S or the M phase was affected.

We thank the reviewer for this valuable comment. Our data so far showed that the very first spindle extension, and therefore M-Phase, is clearly affected (Fig. 4A-B). If the first division fails all subsequent S phases and M phases might be affected at the population level. To test whether S-phase is affected we now acquired time lapse imaging of single cells labeled with the quantitative DNA dye 5-SiR-Hoechst and saw no difference in DNA signal increase for PfSlp KD parasites, while nuclear number was reduced, showing directly that M phase rather than S-Phase is affected (Fig. 4C, lines 280ff).

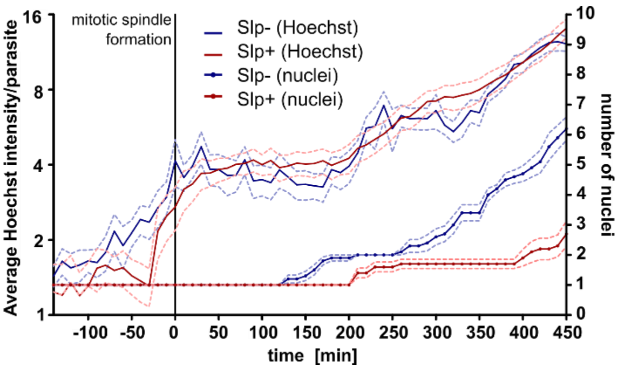

DNA quantification graph (Fig. 2D) is confusing and does not correlate with the quantification of merozoites (Fig. 2E). Why is the DNA intensity of Slp- parasites lower than the DNA intensity of the Slp+ parasites, even though Slp deficient line produces less progeny? Is it possible that you missed the actual peak of DNA replication? Authors may consider more tight time courses with a few additional time points.

This is a good point. We have repeated this experiment with longer sampling time and shorter intervals. We now plot the fraction of cells with DNA content above 2N (also to exclude double infections and cells that arrest prior to the schizont stage) as a measure to see how many cells are replicating (Fig. 2D, lines 175ff). Although the replication peak was, as observed before, shifted by GlcN treatment we found no significant differences in height. Although the lack of PfSlp tagging and GlcN treatment in the 3D7- control might favor the slightly more productive replication. We complement this analysis by plotting the average DNA fluorescence intensity over time (Fig. S7A) and the area under the curve (see below), as an approximation of “total replication activity” and still found no significant differences (Fig. S7B). The fact that the DNA fluorescence intensity peak does not correlate with the slightly reduced merozoite number observed in Fig. 2E is not very surprising as the fixed time point sampling for DNA quantification can’t differentiate between cells slowing or even halting progression and thereby confounding the averages. This limitation of single timepoint population analysis specifically highlight the importance of our time resolved single cell analysis presented later in Fig. 4, which clarifies the phenotype. Further, merozoite

number counting does not give any insight about ploidy of the individual merozoites. Considering the significant nuclear division defect we also show in Fig. 4 it is plausible that some merozoites in the Slp KD could be polyploid, while globally replication is not strongly affected.

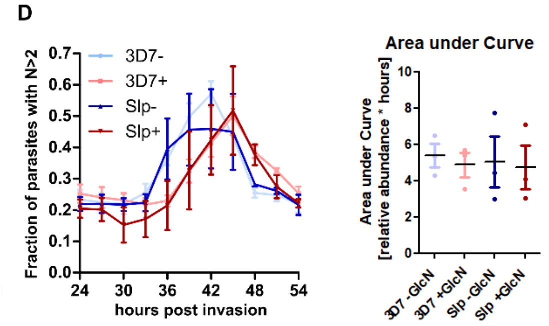

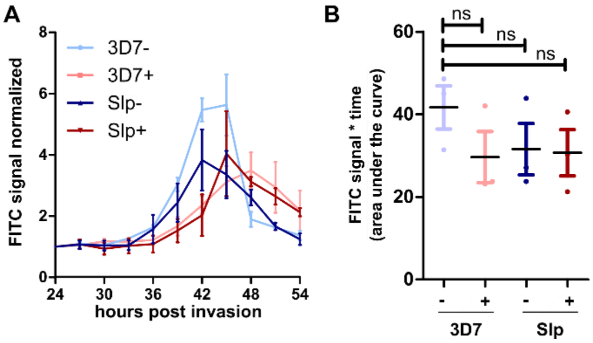

Given the main claim, the study lacks the spatial-temporal analysis of tubulin described only in words. The tubulin quantifications by WB (Fig. S6) are not convincing, as well as the resulting conclusion of the cell cycle retardation.

We are not completely sure what the reviewer is indicating by a lack of spatial-temporal analysis of tubulin given that we show time-resolved imaging data of tubulin organization in dividing cells and quantify intranuclear tubulin levels. Those data (particularly Fig. 4A) clearly show a retardation in the mitotic spindle stage. We, however, acknowledge that the data on tubulin quantification via western blot could, as Reviewer 2 also points out, be improved through the addition of biological replicates. We have repeated those experiments twice and can now confirm by statistical analysis that total tubulin, aldolase, and centrin protein levels are not affected by Slp KD at 24, 30, and 36 hpi (Fig. 3E, Fig. S8, lines 232ff). This indicates that the increase in intranuclear tubulin is not a consequence of globally increased tubulin expression.

It is unclear how the authors arrived at the conclusion that the mitotic spindle is deficient in PfSlp KD parasites. Fig. 3C does not show visible differences in GlcN treated and untreated parasites.

PfSlp KD parasites show unusual microtubule protrusions branching of the main microtubule mass, which have never been observed in wild type parasites. This should have been indicated more clearly by adding an arrow in Fig. 3C. We further think our observation that the tubulin content in mitotic spindles is almost three times higher on average than in wild type spindles (Fig. 3D) and that those spindles do not properly extend (Fig. 4A-B) justifies this claim.

How many nuclei are in the cells shown in figure 4 and supplemental movies? It seems as if GlcN treated Slp parasites form one long spindle.

In a previous study (Simon et al. 2021, LSA, Fig. 1B) we have demonstrated that the number of distinct microtubule foci, i.e. mitotic spindles, observed in cells corresponds directly to the number of nuclei. Hence we can assume that prior to successful spindle extension in the PfSlpKD there is one nucleus or two nuclear masses that are in the process of separation. We now added some new time-lapse microscopy data of DNA- and tubulin-stained parasites that confirms that arrested Slp KD parasites fail to properly divide their nuclei (Fig. 4C, Mov. S4-5) and confirms our previously published findings about nuclear number.

A majority of PfSlpKD parasites indeed seem to form one long spindle. However, this "long spindle" appears only after a significant time delay during which wild type parasites already have undergone multiple nuclear divisions and could be a downstream effect of this retardation through e.g. increase of total tubulin levels over time (Fig. 3E).

The conclusion of anaphase block is unsupported: the authors need to demonstrate the accumulation of the metaphase nuclei with a bipolar spindle.

Anaphase describes the phase of chromosome segregation and includes the full extension of the spindle, as discussed above, both of which fails in more than half of the PfSlpKD parasites (Fig. 4A, Mov. S3, S5) and is therefore interpreted as "failure to properly progress through anaphase" for the first time in the discussion (line 381). We currently can't think about a more direct way to demonstrate this than by time lapse imaging of the very first mitosis in individual parasites. Any analysis of populations at later time point or using fixed cells will be skewed by the phenotype occurring in the very early stages of nuclear division.

Reviewer #1 (Significance (Required)):

The eukaryotic centrosome is a microtubule organizing center that guides the segregation of duplicated chromosomes. Despite being an essential regulator of the parasite division, the apicomplexan centrosome remains poorly understood. Recent studies in Toxoplasma gondii (Suvorova et al., 2015) and Plasmodium species (Simon et al., 2021) demonstrated high diversity of the centrosome organization making the studies of microtubule organizing centers in apicomplexans, particularly challenging. Examining the protein composition is one of the ways to uncover organelle function. The current study would help to understand the evolution of the MTOC and mechanisms of cell division in understudied eukaryotic models.

The focus of my research is the apicomplexan cell cycle. I previously showed the bipartite organization of the Toxoplasma centrosome and identified and characterized several centrosomal constituents, including centrin partner Sfi1. Our most recent study presented evidence of the functional spindle assembly checkpoint in Toxoplasma tachyzoites.

Reviewer #2 (Evidence, reproducibility and clarity (Required)):

Summary:

Plasmodium falciparum parasites undergo several rounds of asynchronous nuclear divisions to produce daughter cells. This process is controlled by the centriolar plaque, a non-canonical centrosome that functions to organize intranuclear spindle microtubules. The organization and composition of this microtubule organizing center is not well understood. Here, Wenz et al. identify a novel centrin-interacting protein, PfSlp, that, following knockdown, leads to fewer daughter cells and aberrant intranuclear microtubule homeostasis and organization.

Wenz et al. identify PfSlp via co-immunoprecipitation of P. falciparum 3D7 strain with an episomally expressed PfCen1-GFP, noting PfSlp as a gene of interest based on the presence of

several centrin-binding motifs. The authors go forward to generate a transgenic 3D7 strain, equipping PfSlp with GFP and glmS ribozyme, to localize and evaluate the function of PfSlp in asexual blood stage parasites. PfSlp appears to, using immunofluorescence and STED microscopy, localize to the outer centriolar plaque in schizonts, based on its colocalization with PfCen3. The authors show, utilizing the inducible glmS ribozyme knockdown system, that PfSlp is required for proper parasite growth, noting a defect following addition of GlcN. This defect is noted to cause a delay in the initiation of nuclear division, or schizogony. Analysis of intranuclear microtubule dynamics reveal abnormal microtubule organization, specifically an increase in nuclear microtubule abundance and length following PfSlp knockdown. Together, these findings characterize the role of a novel protein, PfSlp, that contributes to nuclear tubulin homeostasis and organization during schizogony.

Major comments:

The major claims made by Wenz et al. are largely convincing with the data provided.

1. One area that requires additional attention is the following: Wenz et al. claim PfSlp and centrin to be interacting partners based on 1) co-immunoprecipitation (without prior protein crosslinking), 2) the presence of centrin-binding motifs in PfSlp and 3) colocalization of PfSlp and PfCen3. This interaction is not interrogated fully and claims specific to this point need to be clarified and described as preliminary. As it is written, Wenz et al. claim PfSlp is required for centrin recruitment to the centriolar plaque but this is not investigated fully. The data show lower levels of endogenous centrin at the centriolar plaque in PfSlp knockdown parasites but centrin protein levels are similar in wildtype and knockdown PfSlp parasites. As is, the phenotype attributed to PfSlp knockdown could be attributed to PfSlp or aberrant centrin recruitment to the centriolar plaque. Experiments manipulating PfSlp centrin-binding motifs would strengthen these claims and elucidate the role of PfSlp apart from centrin. If not included, less emphasis should be placed here.

We agree with the reviewer that additional evidence to demonstrate the direct interaction between PfSlp and centrin would be adequate. Due to the presence of multiple widely spaced centrin binding motifs in PfSlp, which would require multiple highly challenging rounds of genome editing to be modified, we have opted for reciprocal co-IP using PfSlp-GFP (line 139, Fig. S3, see below). The exceptionally large size of PfSlp of 407 kDa and low expression prevented us from detecting it directly on the western blot, but we found a clear centrin band in the Slp IP that was absent in the control.

We have also further qualified our formulation about centrin recruitment depending on PfSlp (lines 138, 146). Finally, we agree that there are many factors downstream of PfSlp that can contribute to the observed phenotype, which might include centrins and will be subject of future investigations.

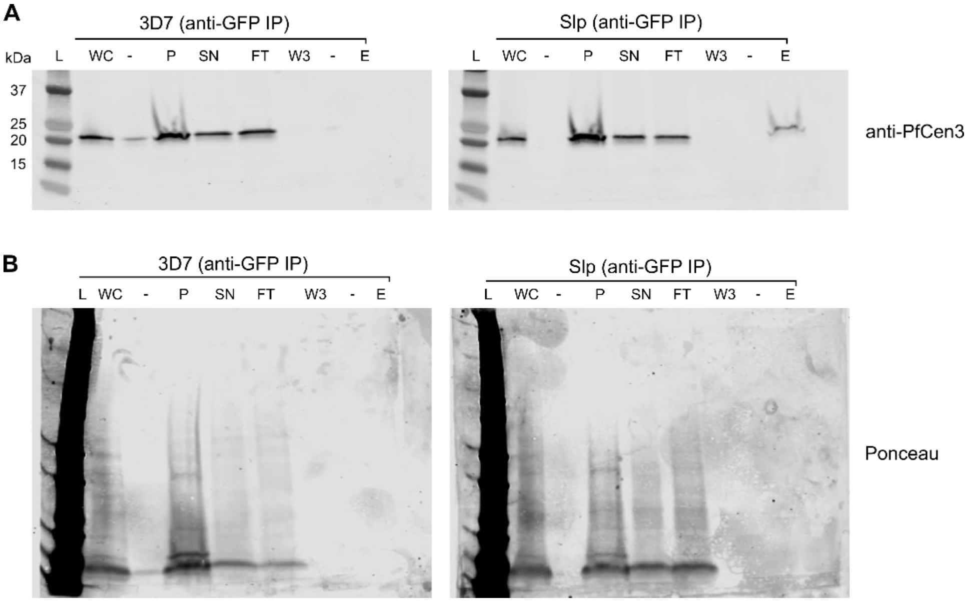

2. The 3.5 mM glucosamine has some toxicity in the parental 3D7. Is it possible to use a lower concentration so the growth of 3D7 is unaffected but the grow of the Slp-GFP GlmS parasites is still reduced?

We acknowledge that the used Glucosamine concentration is on the higher end of the classically used range. The slight toxicity of Glucosamine is dose-dependent and only vanishes at submillimolar concentrations. During initial experiments we have found to generate a robust phenotype with 3.5 mM and decided to carry out all experiments at this concentration. We think that the added effect of PfSlpKD over GlcN treatment alone is sufficiently show as e.g. the merozoite number phenotype (Fig. 2E) and the mitotic delay (Fig. 4B) only occurs in Slp+ parasites.

3. Fig 3E - the quantification of tubulin levels requires biological replicates to have means and error bars.

We fully agree with reviewer 2 (and reviewer 1 who commented along the same lines) and now generated two more biological replicates that allow us to confirm by statistical analysis that total tubulin, aldolase, and centrin protein levels are not affected by Slp KD at 24, 30, and 36 hpi (Fig. 3E, Fig. S8, lines 235ff).

4. The use of "centrin" is somewhat imprecise throughout. The authors should specific which centrin (PfCentrin1 or PfCentrin3 or others) they are referring to each time in the text.

Thank you for requesting this clarification. We have used “centrin” on purpose but have failed to properly explain our terminology in the text. For the detection of endogenous centrin we use a polyclonal antibody raised against PfCentrin3 (Simon et al. 2021). Due to the very high sequence identity between PfCentrin1-4 we can’t exclude cross-reactivity of any polyclonal antibody. Throughout the field so far polyclonal antibodies raised against Chlamydomonas centrin and

Toxoplasma centrin 1 have been successfully used to label centrin pool at the centriolar plaque. Since we can't distinguish with certainty which of the centrins (PfCen1-4) is targeted we chose the general description "centrin". We were however able to show that all four centrins (PfCen1-4) colocalize at the centriolar plaque (Voss et al. biorxiv, /10.1101/2022.07.26.501452) and that Plasmodium centrins interact with each other was demonstrated previously (Roques et al. 2019) while the interaction between PfCen1 and PfCen3 was shown in this study. Therefore, this will not limit our conclusions. We now explain this better in the text (lines 132ff) and adjusted the labeling in Fig. 1E.

5. The mention of the cell cycle checkpoint is an interesting and appropriate point in the discussion. However, the discussion of it in the last sentence of the introduction is less appropriate. It should be removed from line 92-93.

We are excited by the prospects of this study to finally be able to investigate the presence of checkpoint induced delays using time-lapse microscopy, but absolutely agree with the reviewer and have removed the statement in the introduction.

Minor comments:

1. Line 50 - "are remaining unclear" should "remain unclear"

Has been corrected.

2. Line 65 - "players" is quite informal. A better word should be selected.

Was replaced with "factors".

3. Line 223 - "were" should be "where"

Has been corrected.

4. The delay in schizogony which is observed following addition of GlcN (Figure S5) may be made more convincing if the experiment is performed hours post invasion rather than hours post treatment. The synchronization of the parasites is in question as it is described in the methods.

We have included this data from our initial exploratory analyses and since it was not central to our argumentation, we choose to add it as supplemental figure. After producing further data, we came to realize that the classical morphological characterization using Giemsa-staining partly mispresents the relevant transition from the pre-mitotic to mitotic stages as the onset of first spindle formation and DNA replication can't be detected. Previous studies have also indicated that parasites which were drug arrested at the trophozoite to schizont transition were morphologically similar to mid- to late schizonts (Naughton and Bell, 2007). In a context that investigates nuclear division phenotypes we feel that this analysis might rather be misleading and that the provided growth assays, DNA replication quantification, and time lapse movies are significantly more informative. Therefore, we have decided to remove the figure altogether. However, we have moved Fig. S7 to Fig. 4 to show the results of the 3D7+GlcN movie quantification in the context of the Slp+/-GlcN results.

5. In general, data presentation is clear and readable. The growth defect observed following GlcN treatment (Figure 2C) could be made more clear with data normalization to emphasize that which can be attributed to PfSlp knockdown and not GlcN.

This is a good suggestion and we have reproduced the initial dataset (Fig. 2C, Fig. S6, see below) and normalized the 3D7 multiplication rate, which shows the effect more directly than the growth curves displayed before, for Slp-tagging and GlcN treatment (“3D7 corr.”). We still found Slp +GlcN to be the only condition to have a significant reduction in multiplication rate in the first cycle after treatment (24-72hpi) with respect to 3D7 control as well as the normalized 3D7 value (“3D7 corr”).

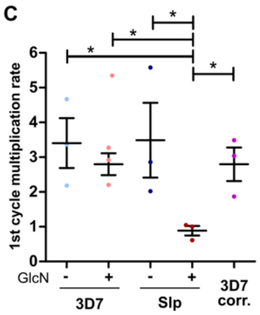

6. Line 276 - Why is nuclear tubulin homeostasis more relevant for closed mitosis? This is difficult to understand. It should be phrased differently or provided with additional explanation.

We thank the reviewer for the comment and agree that this is poorly formulated. We were meaning to express that in e.g. mammalian organisms the nuclear envelope gets disassembled during mitosis and thereby removes the need to regulate import of tubulin into the nucleus for spindle assembly. This is a self-evident statement and has been removed for clarity.

7. Line 316 - "were" should be "was"

Has been corrected.

8. The identity, source, and dilution for each antibody must be reported for each use in the methods.

We noticed that we had not fully referenced Table S3, where we listed all used antibodies and dilutions, which we have now done throughout the methods section.

Reviewer #2 (Significance (Required)):

The mechanisms by which intranuclear microtubule dynamics are regulated by Plasmodium falciparum parasites are not well understood. Furthermore, the proteins that are present near the centriolar plaque remain mostly unknown. Understanding the role of the Plasmodium centriolar plaque and its members is critical to describing these dynamics and contributes to our growing understanding of schizogony, an atypical mode of cell division mode with several rounds of nuclear division lacking cytokinesis. Therefore, the identification and initial characterization of PfSlp1 is useful for malaria parasite cell division community.

Reviewer #3 (Evidence, reproducibility and clarity (Required)):

The work by Wenz and Simon approaches the function of a novel component of the malaria parasite centriolar plaque, a structure whose complexity has begun to be unraveled only recently**, greatly by the same group.** The authors identify a homolog of Sfi1, a centrin binding protein highly conserved in eukaryotes. Sfi1 homologues usually co-localize with centrioles.

As a tool to characterize its function, the authors uses a conditional knock down strategy, based on GlcN addition, to downregulate PfSfi1-like protein (PfSlp). The authors analyze the impact of pfSlp downregulation on cell division progression, and go on detailly characterizing the progression of mitotic nuclear division. In sum the study finds that expression of Slp1 is required for proper progression of cell division in Plasmodium parasites.

The study is well conducted, and the manuscript clearly written. In general terms I found the data shown to support the author's claims. However, I do have a few points of concern to raise, particularly pertaining overinterpretation of the data, and points that need clarification before the manuscript is fit for publication. In particular the authors should explain more clearly how the data based on fluorescence intensity quantifications was acquired and processed, and how this information is intertwined with the expected kinetics of structures measured, along the cell cycle.

We appreciate the positive feedback and the constructive comments made by the reviewer and now adapted our interpretation of the data or provide additional experimental data to strengthen our argumentation as outlined below. Further we have added some detail to the description of our experimental approaches in the methods section.

I outline below major and minor points that require attention,

Major Points

The manuscript stems off the premise that PfSlp interacts with PfCen1. Despite the fact that Sfi1 is a known interactor of centrin, that the identified protein in Plasmodium has centrin binding motifs, and these proteins co-localize, the support for the direct interaction between the two proteins is based solely on the IP/MS result. No reciprocal IP results are shown.

We thank the reviewer for the suggestion and have now added the reciprocal co-IP, which shows a specific interaction between PfSlp and centrin without need for cross-linking (Fig. S3, see also reply to comment 1 by reviewer 2).

Line 118 specifies that co-localization of Slp-GFP with centrin "corroborates their direct interaction." Co-localization most certainly does not show direct interaction. In addition, Figure 1D shows co-localization with Cen3, not with Cen1, which was the only protein shown to have a physical interaction with Slp via immunoprecipitation. Hence, the claim is unplaced and this section should be reworded for clarity.

The reviewer is correct to point out that co-localization even at STED nanoscale resolution does not demonstrate interaction. We have reworded this statement. Cen3 was the only other specific protein found in the Cen1 immunoprecipitation (Table S1) and the interaction between the four centrins Cen1-4 was shown in an earlier study in *P. berghei* (Rogues et al. 2019). However, as the Reviewer 2 also indicated, we did not clearly communicate what the targets of our centrin

antibody are. We, indeed used an antibody raised against PfCen3. Due to the very high sequence identity between centrins it is, however, unrealistic to exclude cross-reactivity between centrins for a polyclonal antibody (as explained in more detail in our response to Reviewer 2). We have added an explanatory statement in the main text (lines 132ff). Our recent finding that GFP-tagged PfCen1-4 all colocalize at the same position in the centriolar plaque (Voss et al. biorxiv, /10.1101/2022.07.26.501452) and our previously published study of the centriolar plaque (Simon et al. 2021) gives us additional confidence that the antibody specifically labels the compartment of interest.

I was surprised to see how little recovery of PfCen1-GFP the authors obtained from their IP experiments. Whilst I understand that a western blot is not quantitative, I wonder, were the amounts of protein loaded onto each lane normalized for comparative purposes in any way? Please comment on this at least in the figure legend so the reader can gage whether the little PfCen1-GFP recovery was a consequence of the IP experiment, or whether the WB is not representative of the actual IP results but rather show a fraction of the recovered material.

We did not determine the total protein concentration (by e.g. Bradford assay) and therefore did not normalize for protein amounts per lane. Instead, we determined the number of infected red blood cells per ml before Saponin-lysis of the red blood cells and loaded protein lysate equivalent to 1×10^7 cells per lane. We now explain this more clearly in the legend for Fig. S1. During the IP, much of the total protein amount might got lost during the washing steps, which might explain the weak Centrin1-GFP band and the absence of a protein signal in the eluate lane by Ponceau staining (neither a signal for Centrin1-GFP nor unspecific protein signal in the Ponceau). We would conclude that the WB, or at least the lane with the eluate, shows a fraction of the recovered material.

If the WB is indeed representative of the actual PfCen1-GFP recovery rates, I suggest you discuss the possible outcomes of having pulled down so little from the total cell lysate - could it be that the recovered proteins are representative of interactions happening only for a subset of soluble PfCen1 molecules? Can the little protein recovery be explained by Cen1 interactions with insoluble cell components such as the cytoskeleton?

As described above, the eluate lane does likely not represent the actual amount of Cen1-GFP that was pulled down and therefore the WB is not representative of the PfCentrin1-GFP recovery rates. Based on our previous studies we are not aware of any cellular PfCen1 pool beside the cytoplasm and the centriolar plaque. Although they might be below the detection limit. The reviewer raises an interesting hypothesis but we don't have sufficient data to assume an association with the cytoskeleton and verifying this would require extended further studies.

Were other IP conditions tested? Were the same results obtained?

We carried out three PfCen1-GFP IPs. Once without cross-linking as shown in the study and twice with cross-linking. The two IPs with crosslinking had different amounts of targets identified (24 vs 162). While we did not detect PfSlp in the one with the low number of peptides we detected PfSlp in the second IP. In both IPs we additionally detected PfCen2 and PfCen3.

Do you get the same interactors if the IP is done using anti-Centrin instead of anti-GFP?

We did not test an anti-Centrin antibody for IPs as the protocol from the Brochet group was optimized for the highly specific bead-coupled anti-GFP antibody.

Please define how you identified "specific hits." This is, please describe your criteria for determining "specificity." Was it an all or nothing selection approach? Are Cen1, Cen3 and PfSlp significantly enriched? And if so, how did you define "enriched for" in the context of your experiment?

We thank the reviewer for given us the chance to clarify our candidate selection. We specifically selected the Cen1-GFP IP targets without cross-linking since it produced a short list of hits detected by mass spectrometry. We used an all or nothing approach in that we subtracted from that list any protein that was ever identified in a GFP control IP analysis by the Brochet lab using the same protocol (Balestra et al. 2021). This left only three proteins Cen1, Cen3, and Slp, as our “specific” hits. We have modified the text to explain our selection criteria more explicitly (lines 112ff) while avoid using the term “enrichment” since this is an all or nothing selection.

I'm not at all suggesting here that you repeat this experiment. I understand that the focus of the manuscript is the description of PfSlp, and this stands regardless of the IP results. However, I suggest you include a lengthier discussion of the results shown in SFig1 and Fig1, and the limitations of the approach.

We appreciate the assessment by the reviewer that the focus of the manuscript is otherwise and acknowledge that this is not an extensive analysis of PfCen1 interaction partners. We have, as requested, added a comment addressing this limitation in the discussion (lines 331ff).

Line 123 mentions that Cen3 and Slp1 are recruited together only because they co-localize in most cells showcasing hemi-spindles. Please simply keep "simultaneously" here, as this is the only thing you can conclude from your quantification data. Being recruited "together" implicitly means by "the same mechanism", which is not shown by your data.

We agree that simultaneously is more accurate and we have modified the text (line 146).

Please specify which statistical test was used for determining significance in Figure S4, and what *** refers to in this case. It is hard to judge really how different these data sets are in light of the overlapping error bars. Also, what is quantified here? Integrated density from an immunofluorescence assay? How are the data normalized to be comparable? How many replicates did you quantify? Or are the data shown representative of a single experiment? I could not find these details in the M&M section or the figure legend.

We have revisited all figure legends and consistently defining the p-value and number of replicates (usually N=3) and briefly explain the measurement. Further we have extended the methods section to make our image quantification approach clearer.

Also, on the interpretation of these data; If Slp1 causes a delay in cell cycle progression, and taking into account that the fluorescence intensity of Slp1 varies along the cell cycle, with Slp1 intensity increasing as cell cycle progresses from the ring stages onwards, are these comparable measurements? In other words, are you selecting the same stages whereby the same Slp1 intensities at the centriolar plaque would be expected?

If I understand correctly these measurements are carried out at 55hs post GlcN addition (when the growth phenotype starts evidencing itself?). At this time point, the relative abundance of ring and trophozoite stages (stages at which Slp1 is not expected to be detectable at the CP) is considerable higher than that of the control condition, hence a reduction in Slp1 is expected, and a mechanistic claim about recruitment or stability would be incorrect. Please clarify.

As the reviewer correctly points out it is important to normalize for the stages when quantifying the PfSlp intensities. To achieve this, we only selected schizont stage parasites with a similar distribution of cells containing 3-10 nuclei between the conditions to ensure we are looking at comparable stages. We then quantified the integrated density at each individual centriolar plaque, designated by the presence of a centrin signal. Outside of centriolar plaques no PfSlp signal can be detected. As for ring and trophozoites stages, they do not have a discernable centriolar plaque, or at least not with the markers available in the field, and likely do not express PfSlp based on published transcriptomics data (Plasmodb.org). We have revisited the text to make our quantification strategy clearer (line 170, 621ff).

To understand the relative contribution of Slp1 to the growth delay phenotype, please include 3D7+GlcN control in the quantification of stages shown in Fig. S5. Please check how the data shown in Fig S5 was normalized; the 49 and 73hs bars in the -GlcN condition exceed 100%.

As indicated in our reply to Reviewer 2 we only included this data from our initial exploratory analyses and since it was not central to our argumentation, we chose to add it as supplemental figure. After producing further data, we came to realize that the classical morphological characterization using Giemsa-staining partly mispresents the relevant transition from the pre-mitotic to mitotic stages as the onset of first spindle formation and DNA replication can't be detected. Previous studies have also indicated that parasites which were drug-arrested at the trophozoite to schizont transition were morphologically similar to mid- to late schizonts (Naughton and Bell, 2007). In a context that investigates nuclear division phenotypes we feel that this analysis might rather be misleading and that the provided growth assays, DNA replication quantification, and time lapse movies are significantly more informative. Therefore, we have decided to remove the figure altogether. However, we have moved Fig. S7 to Fig. 4 to show the results of the 3D7+GlcN movie quantification in the context of the Slp+/-GlcN results.

What is "centrin signal" shown in Figure 2B? Centrin1? Centrin 3? Please clarify which centrin protein you are referring to throughout the manuscript, or provide evidence that they could be interchangeably used for localization and intensity measurement experiments.

We thank the reviewer for pointing out this vagueness. As explained above in the second major point and in the reply to reviewer 2 we use the term "centrin" to emphasize that we cannot be certain to which degree PfCen1,2,3 or 4 contribute to the signal. Our recent preprint (Voß et al. 2022) and Roques et al. 2019 and Simon et al. 2021 however suggest that all centrins co-localize and interact at the outer centriolar plaque. As mentioned we now discuss this in the text (lines 130ff).

Line 149 outlines that Slp1 and centrin intensities are simultaneously reduced, and that this fact alone "affirms" they are part of one complex, and that this implies that Spl1 is somehow involved

in centrin recruitment. This claim is not supported by the data shown. There are multiple possible explanations as to how the intensities of both proteins could simultaneously decrease without them conforming the same structure, the same complex or even directly interacting. For example, if the centriolar plaque homeostasis is altered, or the "intensities" are simultaneously dependent on cell cycle progression, they will both be affected without necessarily ever interacting. In fact, if the centrin intensity monitored is that of Cen3, a direct interaction between Slp1 and Cen3 is not demonstrated at any time. At best, the authors could argue that both proteins are directly interacting with Cen1. Again, even this is no definitive proof that they form the same complex.

The reviewer is correct to point out that there are multiple explanations for the decrease of centrin and Slp signal and we have phrased some of the relevant statements more carefully (lines 138, 146, 172). We, however, think that our new reciprocal co-IP data (Fig. S3) in combination with the already provided evidence now significantly strengthens our claim about the interaction between centrin and Slp.

Measurements of DNA content, shown in Figure 2D, show that +GlcN Slp1 knockdown parasites exhibited reduced DNA amounts at 42hs post induction. These results are interpreted as "defects in nuclear division," however, 1. Nuclear division is not analyzed directly, but rather approximated by measuring DNA content. 2. Even in the presence of perfectly normal nuclear division, the DNA content reduction for these parasites at this time point is expected, as cell cycle progression is affected.

Line 160 states that a reduction in merozoite number corroborates a defect in nuclear division. However, the data shown only quantifies merozoites per schizont. As mentioned above, nuclear division is not directly assayed.

We thank the reviewer for emphasizing this important distinction (alongside Reviewer 1). Making the conclusion about nuclear division based on the reduced number of merozoites was premature and we now phrased this more carefully (line 198). Even our data showing inhibition of spindle extension (Fig. 4A-B), although being a strong indicator, do not strictly speaking observe nuclear division. Hence, we have added time-lapse imaging data of nuclear number in KD vs control conditions using the quantitative live cell DNA dye 5-SiR-Hoechst (Fig. 4C. Mov. 4-5). These data now clearly show that the nuclear division or M-phase is affected, while the increase of DNA signal, which represents replication, is not distinguishable from the control. This confirms that nuclear division is the initial and relevant phenotype.

What the nuclear division defects observed are is unclear. Is there fusion, fission? loss of nuclear content? defects in mitosis completion? defects in DNA replication? A reduction in merozoites per schizont, with a concomitant reduction in overall DNA levels could also be explained by a general arrest in the final stages of division. Do other processes linked to nuclear division progress normally? For example, is there daughter cell formation during schizogony without the expected accompanying nuclear division? Are daughters forming in the correct number and position? Are there more daughter cells than nuclei? Or are parasites dying before completing schizogony and producing merozoites? These possibilities need to be carefully teased out before a nuclear division defect can be assigned as the sole causing factor of the division phenotypes observed.

These are all very pertinent questions some of which go beyond the scope of this very first characterization of PfSlp function but we are keen to include those in our future analysis. Some of them we can answer while I will try to offer an interpretation for the remaining ones:

It isn't fully clear to us what is meant by "Is there fusion, fission". We will assume that the reviewer refers to the process of karyofission where the nuclear membrane is constricted and fused between the segregating chromatin masses. The field is still lacking a nuclear membrane marker, which makes a direct analysis of this question difficult. Under normal circumstances it has been demonstrated that mitosis is fully closed and the nuclei are completely surrounded by membrane right after division (Klaus et al. 2021). To maybe clarify further we use the term nuclear division to designate the formation of two physically distinct nuclei from one progenitor. We can't and don't comment on the integrity of the nuclear membrane and if we had to speculate, it is probably not affected.

Our new data on DNA dynamics (Fig. 4C) shows a delay in nuclear division while DNA replication seems unaffected in the early division stages. The failure to complete mitosis is also shown by the lack of proper spindle extension. It is possible that PfSlp KD affects final stages of division, but since we treat parasites at ring stages and detect a strong phenotype already at the very first division which occurs only a couple of hours after centrin/Slp recruitment one must assume that this is the defining phenotype, which likely has repercussion on later rounds of division. This makes it virtually impossible to clearly define late phenotypes. We actually have to assume that parasites that proceed to later stages of division do so because PfSlp KD was less efficient.

Our data directly shows that more than half of our PfSlp KD parasites "fail to properly divide their nucleus" in the first round of mitosis and therefore can't construe any other way than to designate this as a "nuclear division phenotype". We purposefully don't comment on potential later phenotypes and an impact on cytokinesis (budding) but look forward to investigating this in the future.

Minor Points

• Line 49: consider "...mechanisms remain unclear" instead of "... mechanisms are remaining unclear"

We have corrected this sentence as suggested.

• Readers not familiar with Plasmodium cell division would benefit from having the different stages shown schematically in Figure 1A labeled (ring, merozoite, trophozoite, etc.)

Good suggestion. We have expanded the labeling in Fig. 1A, but still choose to focus on the division stage, which is relevant for the presented data.

• Figure 1 legend: Please specify that "centrin" staining is approximated by centrin 3 specifically. Figure 1E is missing a legend in Figure 1's legend.

Thank you for pointing this out. We have expanded the figure legend accordingly.

• To ease the reader's interpretation of the data, please consider using a different color for 3D7 +GlcN in the plots shown in Figure 2. It is difficult to distinguish the light magenta from the red color at first glance, especially when the lines are partially overlapping.

We explored many different color combinations and consulted with several colleagues and concluded that the chosen color combination is most suitable to convey the logic of the strains (while accounting for green-red blindness).

• Please clarify how long after GlcN addition are phenotypes assessed - ex. Microtubule cumulative length measurements shown in Figure 3.

We mentioned in the previous Fig. 2 that we add GlcN at the ring stage preceding the schizont stage we analyze but failed to specify that we consistently do so for all experiments. We have added more information in the results (line 221) and to the methods section in more detail.

• For Figure 3C please provide a separate image for the Slp channel alone. The overlay of the green centrin signal and the magenta from the tubulin staining render a yellow signal. It is difficult to appreciate the level of Slp knockdown in these cells. Moreover, in the inset, the label "zoom in" is on top of the centrin signal in green, precluding the proper assessment/observation of any yellow signal left over.

Thank you for this remark. We have removed the centrin signal, which is clearly shown in the main panel, from the zoom ins to render the residual PfSlp signal clearly visible.

• When describing Sf1 in T. gondii, please also cite PMID: 36009009 PMCID: PMC9406199 DOI: 10.3390/biom12081115

When submitting our manuscript this study was not yet published, but we are happy to now include it in the introduction (line 92).

The notion of "checkpoint" is mentioned in the introduction and revisited in the discussion. This is a topic under current discussion/evaluation in the field. As mentioned by the authors, demonstration of a checkpoint implies demonstrating reversibility of the putative checkpoint. Though the authors do not make claims about Slp1 or the phenotypes observed activating a specific checkpoint, the manuscript could be further strengthened if the authors showed that the anaphase arrest is reversible upon wash out of GlcN and restored levels of PfSlp expression. I'm including this comment as a "minor points" because it is a only suggestion. I understand that carrying out these experiments is not within the scope of this work. However, if the authors decided to pursue this, it would certainly strengthen the manuscript.

We highly appreciate the suggestion made by the reviewer and already considered ways to inactivate the putative spindle assembly checkpoint or reverse the phenotype. Wash out of GlcN would theoretically be an option although we are unsure that the kinetics of the subsequent protein synthesis would unfold on a short enough time scale. As suggested by Reviewer 2 we try to remain cautious about directly addressing the checkpoint issue, since e.g. PfSlp due to its localization can't be a direct component of the checkpoint itself. The mention of "checkpoints" has also been removed from the introduction. We are, however, excited that using our time lapse microscopy protocols there now is a framework to investigate this in more depth in the future.

Full Revision

Reviewer #3 (Significance (Required)):

Plasmodium species lack centrioles, and display a divergent mitosis. It is therefore of interest and relevance to understand the peculiarities of the centriolar plaque, as it likely underlies the ability of Plasmodium to upscale its numbers.

Our molecular understanding of the underpinning factors controlling nuclear and cell division in Plasmodium is limited to a few recent publications. The data presented herein is novel and contributes to the body of work with molecular insight and high resolution microscopy coming on for the malaria field.

My expertise is in cell division in Apicomplexan parasites
