# Supplementary material for: An Sfi1-like centrin-interacting centriolar plaque protein affects nuclear microtubule homeostasis
Source: PLoS Pathog. 2023 May 2;19(5):e1011325. doi: 10.1371/journal.ppat.1011325 (PMC10180636; doi:10.1371/journal.ppat.1011325)
Supplement: Attachment — Submitted filename: _Response to reviewer comments.pdf [file ppat.1011325.s020.pdf]

Reviewer's Responses to Questions

Part I – Summary

Please use this section to discuss strengths/weaknesses of study, novelty/significance, general execution and scholarship.

Reviewer #1: The study led by Wenz and Simon identified the malaria parasite homolog of Sif1, a centrin-binding protein. They demonstrated that Sif1 interacts with centrin 1 and localizes at the cytosolic compartment of the microtubule organizing center in the Plasmodium falciparum blood-stage parasite. To address Sfi1 function during the parasite intraerythrocytic replication, they generated a conditional knockdown parasite line using the glms Ribozyme system. Next, they employed a combination of super-resolution and live microscopies to demonstrate the critical role of Sif1 in intranuclear homeostasis of tubulin, proper DNA segregation, and parasite growth. The study is well conducted with adequate controls and biological replicates, resulting in conclusive new findings on the atypical Plasmodium cell division mode. The three reviewers from Review commons raised most of my concerns from the preprint version, and the authors' responses satisfied me. I agree with Reviewer 3 comments regarding the additional experiment to test whether Sfi1 is a checkpoint factor that would have strengthened the manuscript by bringing more mechanistic to the more phenotypical description of Sfi1 KD. Lastly, I have a few minor comments for the authors to address before the final publication.

We thank the reviewer for taking the time to re-evaluate our manuscript and their appreciation of our work. Indeed, addressing questions around a potential mitotic checkpoint will be worth pursuing in the future as checkpoint biology is still in its infancy in the Plasmodium field. Our response to the minor comments can be found below.

Reviewer #2: The resubmission of the manuscript by Wenz et al. is devoted to the role of the Plasmodium ortholog of the yeast half-bridge protein Sfi in the parasite cell division. The role of this centrin-interacting protein had not been examined in Plasmodium sp., and, according to the evidence presented in the manuscript, this factor may regulate the first karyokinetic event of the multinuclear division. Although the authors supplemented the revised manuscript with new findings, the study remains limited in scope and needs more experimental rigor. As such, it requires substantial work to support the model of the PfSlp1 function in Plasmodium.

We thank the reviewer for their comments. Our manuscript did not aim to imply that PfSlp is an ortholog of Sfi1 and we made a small text change to highlight this more clearly (line 124). Despite the lack of any sequence homology our aim was to highlight a potential functional resemblance rather than stipulating the presence of a unique centrosomal protein in malaria parasites. Our study uncovered a novel centriolar plaque protein, and we can acknowledge that we did not reveal a complete functional model for PfSlp in this inaugural study. We, however, provide an informative framework to understand the additional functions of PfSlp and those of other centriolar plaque proteins in more detail in future studies.

Reviewer #3: Plasmodium falciparum parasites undergo several rounds of asynchronous nuclear divisions to produce daughter cells. This process is controlled by the centriolar plaque, a non-canonical centrosome that functions to organize intranuclear spindle microtubules. The organization and composition of this microtubule organizing center is not well understood. Here, Wenz et al. identify a novel centrin-interacting protein, PfSlp, that, following knockdown,

leads to fewer daughter cells and aberrant intranuclear microtubule homeostasis and organization.

Wenz et al. identify PfSlp via co-immunoprecipitation of P. falciparum 3D7 strain with an episomally expressed PfCen1-GFP, noting PfSlp as a gene of interest based on the presence of several centrin-binding motifs. The authors go forward to generate a transgenic 3D7 strain, equipping PfSlp with GFP and glmS ribozyme, to localize and evaluate the function of PfSlp in asexual blood stage parasites. PfSlp appears to, using immunofluorescence and STED microscopy, localize to the outer centriolar plaque in schizonts, based on its colocalization with PfCen3. Moreover, PfSlp appears to interact with PfCentrin as evident by western blot analysis following a reciprocal IP using anti-GFP on Slp-GFP parasites. The authors show, utilizing the inducible glmS ribozyme knockdown system, that PfSlp is required for proper parasite growth, noting a replication defect following addition of GlcN. This defect is noted to cause a delay in the initiation of nuclear division, or schizogony. Analysis of intranuclear microtubule dynamics reveal abnormal microtubule organization, specifically an increase in nuclear microtubule abundance and length following PfSlp knockdown. Together, these findings characterize the role of a novel protein, PfSlp, that contributes to nuclear tubulin homeostasis and organization during schizogony.

Major comments:

The major claims made by Wenz et al. are convincing with the data provided. The changes made are satisfactory in response to reviewer comments. Conclusions made about PfSlp and centrins are interesting and strengthened by the addition of the reciprocal IP with Slp parasites. The data presented is clear and biological replicates and proper statistics are present. The discussion of cell cycle checkpoint is interesting and conclusions regarding the impact of this work on this question in the field is not overstated

We thank the reviewer for the compelling summary and the appreciation of our work.

Part II – Major Issues: Key Experiments Required for Acceptance

Please use this section to detail the key new experiments or modifications of existing experiments that should be absolutely required to validate study conclusions. Generally, there should be no more than 3 such required experiments or major modifications for a "Major Revision" recommendation. If more than 3 experiments are necessary to validate the study conclusions, then you are encouraged to recommend "Reject".

All line numbers provided in our response below refer to the manuscript document with tracked changes (and not to the final manuscript files where changes will have been accepted and which is reformatted according to the journal guidelines).

Reviewer #1: None

Reviewer #2: There is a significant discrepancy between the analysis of individual parasites (movies/images) and the bulk (WB, qPCR). For example, there is no change in tubulin expression by WB, while the movies show the multiplication of the tubulin dots in Slp1 expressing parasites.

In general terms western blot, qPCR, and image analysis measure complementary but different cellular parameters. Aside the one example, which we address below, the "discrepancy" is not clearly specified. We will nevertheless attempt to address the different types of data and their relation. Concerning qPCR we only use it to quantify total PfSlp mRNA levels and show a 55% reduction, which matches reasonably well the 40% reduction of PfSlp signal at the centriolar plaque measured by IFA image analysis.

Western blot results for tubulin (Fig 3E), although there is no significant difference in the PfSlp knock down, actually show a significant increase in total cellular tubulin between 24 and 36 hpi, which likely occurs to allow spindle formation in the growing number of dividing nuclei. This is well reflected in the movies where the total intranuclear microtubule (SPY555-tubulin) signal increases over time. The difference between control and PfSlp KD is only detected in the amount of nuclear signal which we can measure by IFA. Tubulin is, however, ubiquitous in the cell and bulk cell analysis cannot specifically interrogate expression in a subcompartment.

The "multiplication of tubulin dots" stems form the normal progression of the parasite through the schizont stage and is a consequence of the multiplication of nuclei which each contain their own mitotic spindle. Our tubulin signal quantification looks at the nuclei as a whole and despite the signal in knock down multiplying significantly less the total signal in the nuclei still increases (above wild type) and therefore provides complementary information to the movies.

This is likely the result of inefficient PfSlp1 knockdown, which was brought to the anthers' attention in the previous submission. A 55% mRNA reduction after 73h with GlcN is not an efficient knockdown for looking at the target protein function.

To the knowledge of the authors there is no definitive threshold for protein or mRNA knock down that is qualified as sufficient, or efficient, in the field. Whether cells react to small or big changes in protein levels is dependent on the protein of choice. An important criterion is whether a knock down generates a specific, reproducible, and significant phenotype, which in our opinion is sufficiently demonstrated throughout the presented data (e.g. Fig. 2C, E Fig. 4B).

Furthermore, the Slp1 tagging/expression should be demonstrated at the protein level. Although the size of the protein is an understandable obstacle, it is a poor excuse for the lack of evidence. There are technologies to enhance the signal (spaghetti monster epitopes, large protein resolution PAGE).

Indeed, we would have liked to present a western blot showing tagged PfSlp. Together with the low expression levels the big protein size indeed presented an "understandable obstacle" in generating these data. Our IFA analysis however does provide an analysis at the protein level. Using antibodies, we can detect a specific PfSlp-GFP signal at the centriolar plaque which is absent in wild type cells and is reduced upon knock down in the Slp strain (Fig 2B). The scope of the project did not allow us to generate, validate, and analyze an entirely new transgenic *P. falciparum* strain containing e.g. tagging with the spaghetti monster epitope.

In line with this concern, how do you set up experiments if the maximum effect is at 73h and the lytic cycle of the parasite is 48h?

The addition of Glucosamine caused a delay in cell cycle progression in the treated cells during the first cycle (Fig. 2D). This difference adds a confounding factor to the analysis of mRNA levels at the population level as the gene we were quantifying significantly increases in transcription during the schizont stage (see Plasmodb entry below). We actually found that in

the second cycle, specifically at around 73 hours post treatment, the +GlcN and control parasite populations were actually more synchronous, possibly due to the most strongly affected parasites dying in the first cycle, and therefore picked this time point for RNA harvesting. The IFA analysis of PfSlp signal (Fig. 2B) was however done in the first cycle since we could easily select a homogeneous population of parasites with about 3-7 nuclei for treated and untreated cells.

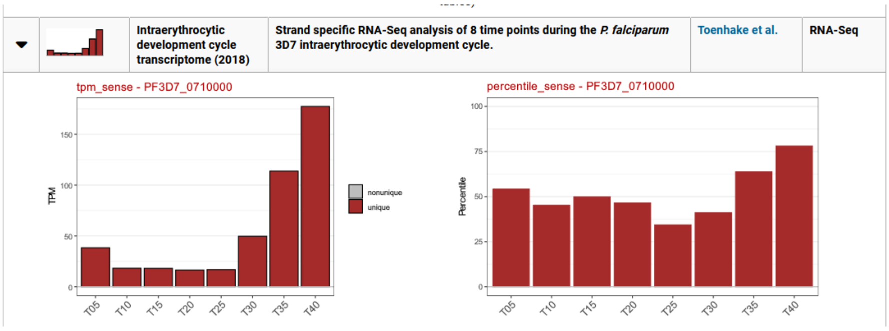

The data interpretation/analysis is questionable. The main conclusion of the PfSlp1 role in mitosis is founded on the results shown in Fig. 4. However, if the graph in Fig. 4C was made of the movies 4-5, then quantifications of the DNA replication do not match what is in video files. At the late time points (~600 min), non-treated parasites have significantly brighter Hoechst intensity (and multiple nuclei) than GlcN-treated parasites (one nucleus). At the same time, the lines of both experimental sets are nearly merged on the graph. Also, the number of tubulin dots is not the proper representation of the number of nuclei: the same nucleus in pre-mitosis has 1 dot, in metaphase 2 dots, and back to 1 dot in anaphase and telophase.

The graph in Fig. 4C was generated by averaging the measurement from 10 individual movies for each condition of which Mov S4 and S5 are two representative examples. For this data series from each movie were aligned to the time point of mitotic spindle formation (i.e. hemispindle collapse). In our quantitative analysis we have omitted later time points beyond 450 min to avoid confounding effects of the potential long term phototoxicity and changes in permeabilization of Hoechst dyes, even when use at only 20 nM. The supplemental movies, however, display the full movie run time, which naturally start at slightly different moments of the trophozoite to schizont transition. We still left those time points in the supplemental movies since we consider them visually informative for the reader. Below we show only the two data series for the movies S4 and S5 which also show little difference in total DNA signal over time and can therefore be regarded as representative. Hence, we conclude that our claims about progression of DNA replication and nuclear division stand.

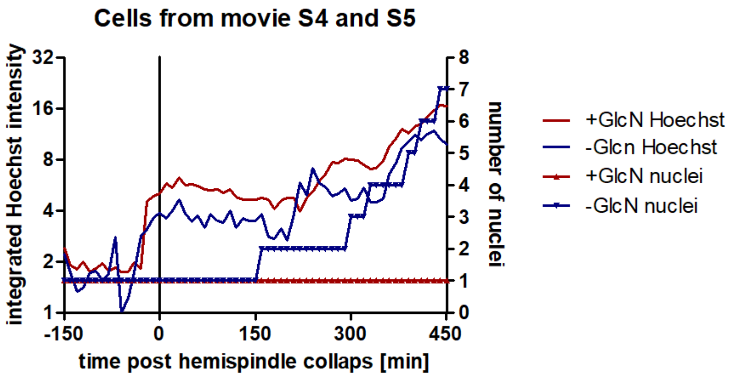

The manuscript needs better organization. At times the writing is redundant and confusing, and the results need to be segregated into sections.

To improve the organization of the manuscript we now provide sub-headings for the individual result chapters.

Reviewer #3: The following are areas that need to be addressed:

• Line 82: You say "cytoplasmic microtubules are absent in schizonts." This isn't true. There is a single spine of subpellicular microtubules in later stage schizonts and fully formed merozoites (see reference). These are widely observed in the field.

o Harding, C. R. & Frischknecht, F. The Riveting Cellular Structures of Apicomplexan Parasites. Trends Parasitol. 36, 979–991 (2020).

Thank you for pointing out this inaccuracy. We aimed to highlight the differences to vertebrate model organisms that have a cytoplasmic interphase microtubule skeleton or yeast that nucleate astral microtubule into the cytoplasm. We are of course aware of the presence of subpellicular microtubules in late schizont and segmenter stage parasites and have now written this more accurately (lines 81ff).

• Line 226-227 AND Figure 3C: You observe tubulin protrusions but do not quantify the frequency at which you observe this in Slp + GlcN parasites compared to your controls. Sometimes parasites just look weird, and quantification of this phenotype will strengthen your claim.

This is a good suggestion. We have looked back at our images and counted 18 out of 40 (45%) spindles in the PfSlp knock down strain to display clear protrusions, while in the control spindles we only detected 6 out of 42 (14%) that might have an aberrant structure. We have added this information to the main text (lines 218ff).

• Line 369-371: You say "the centriolar plaque...raises the possibility that this specialized nuclear pore." This sentence suggests that the centriolar plaque is a nuclear pore – is this what you mean? If so, please give more explanation and clarify.

In our previous study (Simon et al 2021) we showed that the intra- and extranuclear centriolar plaque compartments are connected through a "opening" in the nuclear envelope by a protein dense neck. In our electron microscopy images this neck looked similar, but wider, than a nuclear pore. Whether this neck is a nuclear pore-like structure in terms of composition or something completely different is not clear at this point. We now phrase this more carefully by omitting the term pore (line 361)

Part III – Minor Issues: Editorial and Data Presentation Modifications

Please use this section for editorial suggestions as well as relatively minor modifications of existing data that would enhance clarity.

Reviewer #1: Line 81: The authors wrote that " while cytoplasmic microtubules ae absent in schizonts" knowing that subpellicular microtubules assemble from the apical ring in the parasite

cytoplasm during schizogony, the authors must clarify that they meant that no cytoplasmic microtubules are nucleated by the CP.

As also requested by reviewer 3 we have clarified this statement (lines 81ff).

Line 86: As Simon et al 2021 demonstrated and used, NHS-ester is a CP marker in parasite post expansion microscopy. Therefore I suggest the authors to write" the only currently known extranuclear CP marker in non-expanded parasites".

This is a valid comment. However, NHS-ester is unspecific and requires the observer to integrate many contextual information of the image, depending on the parasite stage, to decide which structure is labeled. Even though centriolar plaques are noticeably more protein dense, in later stages e.g. rhoptries are also very strongly stained by NHS-ester. Therefore we want to suggest the formulation "the only currently known extranuclear CP marker protein" for more clarity (line 88).

I found the data in sup fig 8 essential to support the functional role of Sfi1 in Tubulin homeostasis and would move the data to the main figure 3.

We understand the point of view of the reviewer. We specifically decided to put this figure in the supporting information since the quantifications shown no significant differences. Personally, we think that this way the "flow", understandability, and clarity of the manuscript are improved.

In the discussion section, the authors claimed in line 369: " The positioning of PfSfi1 close to the neck of the centriolar plaque" What are the data supporting this claim and could they define what is the neck of the centriolar plaque. In Simon et al 2021 they refer the CP as a hourglass shape structure and it is unclear what is the CP neck.

Thank you for pointing out the lack of explanation of the term neck, which we casually use to designate the most protein dense region of the "hourglass shape", which specifically spans the nuclear envelope. From the Simon et al. 2021 study we know that centrin, and therefore PfSlp, is close to that region. We have replaced "neck" with the more general designation "region" (line 369).

Reviewer #2: It is unclear why some of the data is included. Lines 185-195 and Fig 2D show no difference between the parent and the PfSlp1 strain treated with GlsN. It does not add to the story but instead makes the story unfocused.

We think that it is important to initially show that GlcN also has a slight unspecific effect on cell cycle progression, which is different from the specific effect produced by PfSlp knock down. This will allow the reader to recognize the advantages and limitations of the used system and contextualize the presented findings.

Reviewer #3:

Minor comments:

• Line 42: Replace "begin" with "beginning"

Has been corrected.

• Line 45: Replace "stage" with "stages"

Has been corrected.

• Line 142: You say "Upon transition into schizogony the schizont stage late trophozoites develop a hemispindle in their nucleus of which about half carry a centrin signal." Include a comma, as follows "the schizont stage late, trophozoites develop"

Has been corrected.

• Figure 1A: Your schematic depicting the first nuclear division labels a 1N parasite (with a hemispindle assembled) as a "schizont." Schizonts, to our understanding, are parasites with 3+ nuclei (see reference).

o Delahunt, C., Horning, M. P., Wilson, B. K., Proctor, J. L. & Hegg, M. C. Limitations of haemozoin-based diagnosis of Plasmodium falciparum using dark-field microscopy. Malar. J. 13, 147 (2014).

This is a reasonable point. We fully acknowledge the classical morphology-based classification of intraerythrocytic parasite stages which includes schizonts as stages with more than two nuclei. We, however, feel that in the light of recent advances in the molecular and cell biological study of the parasite cell cycle we should allow adaptations to the use of the term schizont. We have recently published a review article, which we now cite (line 45), addressing this particular issue (citation and relevant paragraph below). Nevertheless, we agree that the designation of a 1N hemispindle parasite as schizont is "too early" and we have shifted the label to the parasite undergoing S-phase in Fig 1A.

Voß Y#, Klaus S#, Guizetti J*, Ganter M*. 2023. Plasmodium schizogony, a chronology of the parasite's cell cycle in the blood stage. *PLOS Pathog* **19**:e1011157.

"Classically, the distinction between the trophozoite stage and the schizont stage has been made morphologically, and a schizont is often referred to as a multinucleated cell, i.e., a cell with more than two nuclei. While this classification seems intuitive, it appears artificial from a cell cycle point of view. According to the morphological definition, DNA replication and nuclear division occur in both the trophozoite and the schizont stage. Moreover, intrinsic and extrinsic perturbations appear to demarcate a major cell-cycle transition at the beginning of the first S-phase (Box 1) [13–16]. While the classic, morphology-based staging is experimentally easy to accomplish and sufficient for many research questions, it bears limitations as it pools stages that resemble cells in G1 with stages in which S-phase or nuclear division already occurred. Therefore, we encourage, if possible, the use of an alternative, cell-cycle based staging, in particular when investigating the cell cycle of P. falciparum. ***Thus, in the context of this review, we consider a schizont as all developmental stages from the onset of the first S-phase to the conclusion of merozoite formation."***

• Figure 1D: Avoid the word "zoom" when referring to your STED images. It is confusing and may lead readers to think these are digital zoom-ins of your confocal images rather than separate STED images.

We have removed the "zoom" label from the panels and adjusted the legend.

• Line 277: You mention a “mitotic spindle phase duration” but do not write the value in the text as it is written. Would be nice to include.

The time values have now been added to the main text (lines 269ff).

• Figure 4D: Include color labels in your figure legend.

Thank you for pointing this out. We have added the color labels to the legend.
